# Novel Pancreatic Endocrine Maturation Pathways Identified by Genomic Profiling and Causal Reasoning

**DOI:** 10.1371/journal.pone.0056024

**Published:** 2013-02-13

**Authors:** Alex Gutteridge, J. Michael Rukstalis, Daniel Ziemek, Mark Tié, Lin Ji, Rebeca Ramos-Zayas, Nancy A. Nardone, Lisa D. Norquay, Martin B. Brenner, Kim Tang, John D. McNeish, Rebecca K. Rowntree

**Affiliations:** 1 Pfizer Regenerative Medicine, Cambridge, Massachusetts, United States of America; 2 Pfizer Regenerative Medicine, Cambridge, United Kingdom; 3 Pfizer Computational Sciences Center of Emphasis, Cambridge, Massachusetts, United States of America; 4 Pfizer Cardiovascular and Metabolic Diseases, Cambridge, Massachusetts, United States of America; Wellcome Trust Centre for Stem Cell Research, United Kingdom

## Abstract

We have used a previously unavailable model of pancreatic development, derived *in vitro* from human embryonic stem cells, to capture a time-course of gene, miRNA and histone modification levels in pancreatic endocrine cells. We investigated whether it is possible to better understand, and hence control, the biological pathways leading to pancreatic endocrine formation by analysing this information and combining it with the available scientific literature to generate models using a casual reasoning approach. We show that the embryonic stem cell differentiation protocol is highly reproducible in producing endocrine precursor cells and generates cells that recapitulate many aspects of human embryonic pancreas development, including maturation into functional endocrine cells when transplanted into recipient animals. The availability of whole genome gene and miRNA expression data from the early stages of human pancreatic development will be of great benefit to those in the fields of developmental biology and diabetes research. Our causal reasoning algorithm suggested the involvement of novel gene networks, such as NEUROG3/E2F1/KDM5B and SOCS3/STAT3/IL-6, in endocrine cell development We experimentally investigated the role of the top-ranked prediction by showing that addition of exogenous IL-6 could affect the expression of the endocrine progenitor genes NEUROG3 and NKX2.2.

## Introduction

Diabetes is a highly prevalent disease characterised by elevated and poorly regulated blood glucose caused by a defect in insulin production by the pancreatic beta cell, reduced insulin action in its target tissue, or a combination of the two. The World Health Organisation estimates that diabetes currently affects 220 million individuals worldwide (http://www.who.int/mediacentre/factsheets/fs312/en/) rendering this a huge area of interest for the medical and drug discovery fields. Over a decade ago, Shapiro and coworkers demonstrated a pathway to a cure by restoring glucose control via the transplantation of pancreatic islets from cadaveric donors into diabetic patients [1]. However, this method is hindered by the scarcity of donor material [2], resulting in intense scientific interest in the generation of renewable sources of pancreatic islet cells for cell replacement therapy.

A major advancement toward this goal was achieved by D’Amour and colleagues [3] when they developed a high-efficiency method of converting pluripotent human embryonic stem cells (hESC) into pancreatic endocrine cells. This was accomplished by using a precise, stepwise combination of growth factors and small molecules to recapitulate *in vitro*, the developmental biology of the human pancreas. They further demonstrated that while these cells are minimally functional *in vitro*, transplantation of the cells into recipient mice allows for their complete maturation into functional glucoregulatory islet-like cell clusters [4].

While this work has been a major advance in the field of islet cell therapy, an added advantage of this cell differentiation system is that it provides experimental access to a heretofore unavailable window of human embryonic pancreas development, namely from germ layer specification through to pancreatic endocrine cell commitment. Although the entire time-course of developmental steps is of general interest, we are particularly interested in the molecular events leading to endocrine cell commitment and differentiation. We hypothesized that understanding the genetic control of early islet cell development and differentiation would reveal novel pathways regulating endocrine cell function in the adult.

To improve the understanding of these molecular events three levels of biology were probed at a genome-wide scale: gene expression, miRNA expression and epigenetics. Similar techniques have been used to study *in vitro* developmental processes in a range of directed differentiation stem cell-based models [5] including the generation of neural cells [6], intestinal tissue [7], adipocytes [8] and myoblasts [9] as well as islet production itself [10], [11]. Outside the context of directed differentiation, mature mammalian beta cells and islets have also been extensively profiled at the epigenetic [12]–[14], miRNA [15]–[17], protein [18] and gene expression levels [19]–[22]. Even with the availability of this extensive background literature, the efficiency of the directed differentiation protocol we use and the integration of three different genome-wide datasets results in unique insights into the formation of pancreatic endoderm.

One of the key goals of this analysis is to identify novel regulators of the latter stages of pancreatic endoderm formation, as we hypothesise that some of these regulators may be manipulated as novel targets for the treatment of diabetes. The identification of such causal drivers of biological processes is a crucial task in many drug discovery projects. High-throughput techniques, such as microarrays and next-generation sequencing, are limited in that they only measure the response of a cellular system. They do not, however, address the key question of unraveling the causal cascades of signaling molecules, receptors, kinases and transcription factors that lead to the observed response. We use an innovative causal reasoning approach (known as the Causal Reasoning Engine (CRE)) that leverages prior biological knowledge, available in published literature, to identify putative novel regulators and regulatory pathways involved in endocrine pancreas development.

We show that hypotheses generated using CRE algorithms can be borne out by laboratory testing in our pancreatic precursor model system. As evidenced by the predicted role of IL-6 in the promotion of endocrine cell formation, we show that addition of exogenous IL-6 to cells at the pancreatic precursor stage resulted in an increase in NKX2.2 and NEUROG3 expression, indicative of new endocrine specification, validating the approach and providing a number of new potential targets for exploration.

## Results

### Directed Differentiation of hESC to Endocrine Precursors

In an effort to explore the molecular pathways involved in pancreatic endocrine cell formation and maturation, we turned to the Viacyte hESC directed differentiation cell model. This system has been previously reported to be capable of generating pancreatic progenitor cells that can fully differentiate into functional insulin-producing cells upon implantation into mice [4]. We reasoned that this *in vitro* culture system, while not an identical surrogate of *in utero* human pancreatic development, should recapitulate many of the critical cell fate decisions occurring during pancreatic organ development, and do so in a more experimentally tractable format. As a first step, we internalized a modified protocol developed by Viacyte to perform their cell differentiation in a non-adherent rotating culture format [23]. Using this improved method, we could increase cell yield while maintaining 90–99% purity at each stage (Figure S1(A)). The synchronicity in differentiation is maintained up until the cells enter the pancreatic lineage, when the culture becomes a complex mixture of pancreatic progenitors and committed endocrine cells as the cells undergo their final differentiation steps and select their ultimate cell fate (duct, acinar, or endocrine cells).

The complexity of the cell population generated can be seen by flow cytometric analysis at day 14 (Figure S1(B)). By this stage, approximately half of the cells in the culture are committed endocrine cells, by virtue of their expression of the pan-endocrine lineage marker Chromogranin A. This ChromograninA population consists of a mixture of cells expressing hormones from each of the differentiated lineages (α, β, δ, ε, PP) (Figure S1(A) and S1(B)). A low level of off target differentiation into intestine, liver, anterior endoderm or mesoderm was seen. Pancreatic progenitor cells, defined by co-expression of NKX6.1 and PDX1 and the absence of ChromograninA, typically comprise between 20%–40% of the culture. In a confirmation of the previous report [5], we showed that when these cells were implanted into the epididymal fat pad of SCID-bg mice, they were competent to mature into functional islet-like cell. Glucose-dependent human C-peptide secretion was observed by 8 weeks post-implant, with maximal function reached by 6 months (Figure S1(C)) indicating that the grafts are functional and that the *in vitro*-generated cells have pancreatic endocrine progenitor properties. Using this model system as a platform, we sought to further understand the gene expression changes that contribute to pancreatic endocrine cell formation. The high cell purity, coupled with the synchronous response of these cells to the inductive cues provided in the protocol make it particularly amenable to detailed transcriptome analysis at each stage from pluripotent cell through the early endoderm and foregut stages.

As a proof of concept that relevant pathway information can be identified using this model, we first sought to validate the system by examining changes in mRNA and miRNA expression as well as global methylation patterns within the early stages of the differentiation. This is a period in which there is an exceptionally high degree of homogeneity in the culture, which facilitated the interpretation of the data.

Samples were harvested at the end of the pluripotent hESCs (day 0), mesendoderm (day 1), definitive endoderm (day 2) and primitive foregut (day 5) stages from three independent differentiation experiments. Additional samples were harvested at days 8 and 11 as these cells develop through the pancreatic progenitor and endocrine lineage. Samples were then profiled for whole genome gene and miRNA expression analysis using Illumina Human HT-12v3 and Human v2 miRNA BeadChips respectively. In parallel, additional cell samples were processed for analysis by ChIP-Seq using an antibody against histone 3 lysine 4 trimethlyation (H3K4me^3^), an epigenetic mark of active chromatin.

### Comparison of Global mRNA, miRNA and H3K4me^3^ Time-course Profiles

**Figure 1:**
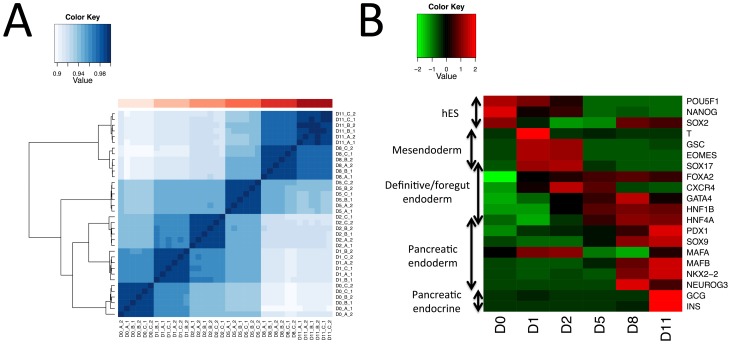
Transcriptome analysis shows the reproducibility of the differentiation protocol. (A) Genome wide gene expression correlation heatmap between samples. Samples are clustered by the Euclidean distance between rows/columns and single linkage clustering. The colored bar along the top of the heatmap indicates the timepoint at which the sample was taken (pink: day 0, maroon: day 11). (B) Heatmap of expression of selected markers. The developmental stage is indicated by the labels to the left of the heatmap.

As an initial analysis to demonstrate the consistency of the system across independent experiments, we generated a clustered heatmap based on the correlation of global gene expression levels (Pearson correlation coefficient (R)) between samples Figure 1(A). The samples from each time point cluster closely together (the minimum R between two samples from the same time point is 0.99), demonstrating the highly reproducible nature of this protocol. Similarly, we generated heatmaps from the miRNA and H3K4me^3^ data (Figs S2(A) and S2(B)), with the same patterns of clustering seen in both cases. Principal component analysis (PCA) plots of the same data that show similar patterns of clustering are also given in the supplementary materials (Figure S3).

The microarray generated gene expression data was confirmed by spot-checking the expression of 113 selected genes (listed in Table S1) by qRT-PCR. The correlation between the measured fold changes across time from the two technologies was high, with 88% of transcripts showing a correlation with R >0.7 and 82% with R >0.9. The relative expression levels by array and qRT-PCR for four exemplar genes and a histogram of all correlation coefficients are given in the supplementary materials (Figures S4 and S5). Figure 1(B) shows the expression profiles for a set of commonly used markers at each stage of the protocol. The expression patterns of these markers confirm the correct production of the relevant cell type at each point in the protocol. The top 20 differentially expressed (P<0.01; fold change >1.5; ranked by P) genes and miRNAs at each time interval are provided in the supplementary tables S2 and S3.As well as gene markers, a number of miRNAs are known to be involved in pancreatic development and insulin secretion. Our data support a role for some of these species in the *in vitro* differentiation process examined here. We observe large increases in expression over time for miR-375 [24], [25], miR-7 [26] and miR-503 [15], though for the insulin secretion regulating miRNA miR-9 [27] expression rises and then falls (data not shown). A recent study by Bolmeson *et al.*, of miRNA enriched in pancreatic islet samples versus liver and skeletal muscle identified miR-127-3p, miR-184, miR-195 and miR-495 [28]. Although our analysis does not extend to include fully functioning islets, we did detect increased expression of miR-127-3p and miR-495 at day 8 and day 11. miR-184 was also detected at high levels at day 11 relative to the day 1– day 8 samples, but we also observed high expression in the starting hESC population, suggesting that although it may have a role in development it is not islet-specific. Finally, in contrast to the observations by Bolmeson *et al.*, our data reveals a strong reduction in miR-195 expression during differentiation. This could possibly be due to donor variation, given that our data is generated from a single hESC line as opposed to a pool of donors, or indicate that the trend reverses later in differentiation.

### Correlations between H3K4me^3^ and mRNA Expression

A high level of H3K4me^3^ is generally considered a marker of actively transcribed genes [29]–[31]. Using the MACS tool [32] we found a relatively consistent number of significant H3K4me^3^ peaks across the time course, though there was a slight downward trend observed. The number of peaks detected ranged from ∼38,000 at day 0 to ∼32,000 at day 11. As has been observed previously [33], in our data H3K4me^3^ peaks tend to overlap with transcriptional start sites (TSS) and the levels form a characteristic double peak (Figure S6).

**Figure 2:**
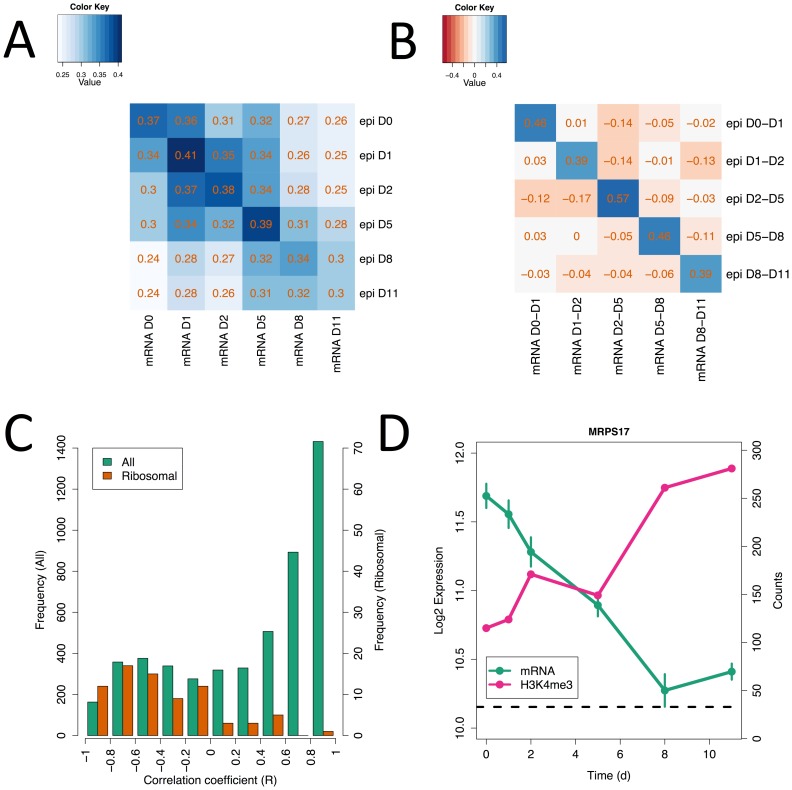
Epigenetic changes generally correlate with transcriptional changes. (A) Heatmap of the correlation between H3K4me^3^ levels (rows) and gene expression (columns) at every pair of timepoints. (B) Heatmap of the correlation between changes in H3K4me^3^ levels (rows) and changes in gene expression (columns) at every pair of time intervals. (C) Histogram of the correlation coefficients (R) for the correlation between H3K4me^3^ levels and gene expression across time for every gene in the dataset. Green bars show the distribution of R for all genes and red bars show the distribution for ribosomal genes only. (D) Gene expression (green) and H3K4me^3^ levels across time for ribosomal gene MRPS17.

Next, we tested to see whether the H3K4me^3^ levels around each TSS correlated with gene expression levels. A positive correlation between absolute H3K4me^3^ and gene expression levels is observed for all time points, though it is weaker at some of the later points and at day 11 in particular (Figure 2(A)). A stronger effect is seen when the *changes* in H3K4me^3^ and expression levels between consecutive time points are correlated. A matrix of the correlations between all possible pairs of time intervals is shown in Figure 2(B). This analysis shows that the expression changes between any two consecutive time points are well correlated with the H3K4me^3^ changes in the same time interval, and that no correlation is observed with gene expression changes at subsequent (or preceding) time intervals. This implies that, at this time resolution, H3K4me^3^ levels around a TSS are not predictive of future expression levels, but rather tend to reflect the degree of transcriptional activation at the site at the moment of sampling.

We also examined whether the correlation between H3K4me^3^ levels and transcriptional activity was better for some groups of genes than others. Figure 2(C) shows a histogram of the correlation coefficients between H3K4me^3^ levels and expression levels calculated for each gene across the time-course. A clear peak towards the right hand side can be seen, consistent with the view that H3K4me^3^ positively correlates with expression. However we were surprised to see a small, but significant bimodality in the data (P<1e−3, Hartigan’s Dip Test), with an apparent secondary hump in the distribution of correlations around R = −0.5. To understand if the genes that showed a negative correlation between H3K4me^3^ levels and gene expression levels corresponded to any particular functional grouping, a gene set enrichment test was run using the correlation coefficients as the basis of the gene ranking. The result suggests that, of all functional classes, ribosomal genes show a significant degree of anti-correlation between H3K4me^3^ levels and gene expression. The distribution of correlation coefficients for ribosomal genes is shown in Figure 2(C) in red alongside the genome wide distribution in green. The expression and H3K4me^3^ levels of an exemplar ribosomal gene are shown in Figure 2(D).

**Figure 3:**
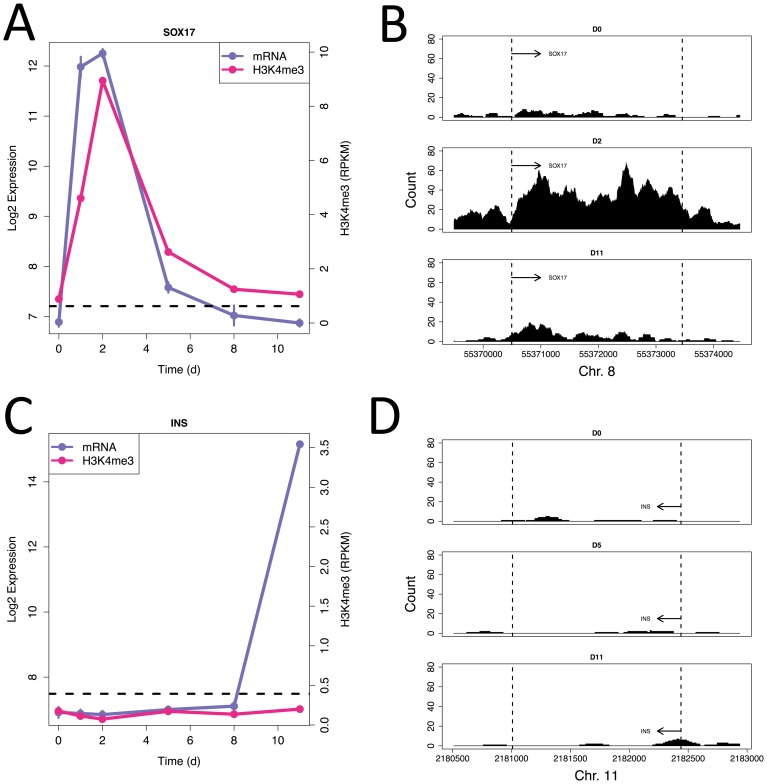
Induction of insulin expression is not accompanied with epigenetic changes. (A) Gene expression (blue) and H3K4me^3^ level (red) profiles for SOX17. The horizontal dashed line indicates the background H3K4me^3^ level. (B) H3K4me^3^ reads piled up over the SOX17 gene body at days 0, 2 and 11. The start and end points of SOX17 are indicated by dashed lines. (C&D) As for (A&B) but for Insulin.

SOX17, a definitive endoderm marker, is more typical in showing a strong peak in H3K4me^3^ levels around its TSS at the same time points as its gene expression peaks (Figures 3(A) and (B)). However, a recent study [14] showed that pancreatic islets have low levels of H3K4me^3^ around the genes encoding secreted hormones such as insulin and glucagon, despite these cells showing high expression of these genes. We observe the same effect in our day 11 sample, where the expression of insulin is induced without any concomitant increase in H3K4me^3^ levels around the insulin TSS and within the gene body (Figure 3(C) and (D)). We observe a similar trend for a number of other genes expressed specifically in the day 11 sample including ISL1, and the secreted peptides GHRL and SPP1. This suggests that transcriptional regulation without concomitant changes in H3K4me^3^ levels is a not uncommon feature in the latest stages of differentiation; consistent with our earlier observation that the correlation between H3K4me^3^ and expression is weakest at day 11.

Across the whole genome, our H3K4me^3^ profiles from the day 11 sample showed good correspondence with those derived by Stitzel *et al*. from islet cells, with 87% of the peaks overlapping. Of the peaks that are unique to the day 11 cells in our dataset (not observed at any of the other time points) 39% overlapped with the islet cell peaks, compared to 22% for the peaks unique to the day 0 cells.

### miRNA – Gene Regulatory Interactions

In contrast to the H3K4me^3^ data, the integration of miRNA data with gene expression data is complicated by the fact that miRNAs can be associated with the regulation of multiple genes and many of these associations are only based on *in silico* predictions.

To address this, we use a pragmatic approach whereby we combine known, literature curated miRNA – gene associations from Tarbase [34] with confident *in silico* predictions – defined as associations predicted by two or more of the targetscan [35], PicTar [36] and mirbase [37] databases. Since miRNAs generally function through inducing the targeted degradation of their target mRNA [38], we then filter these associations by only considering those where the expression levels of the gene and miRNA are anti-correlated. Not all miRNA – gene interactions are repressive at the transcript level [39], so this may lose some true functional interactions, but it reduces the number of associations to a more manageable level. As a second level of filtering, we also consider the H3K4me^3^ levels of the target gene. This allows us to filter out those genes where the changes in gene expression level are adequately explained by the H3K4me^3^ level and so parsimony dictates that miRNA regulation is not required.

**Figure 4:**
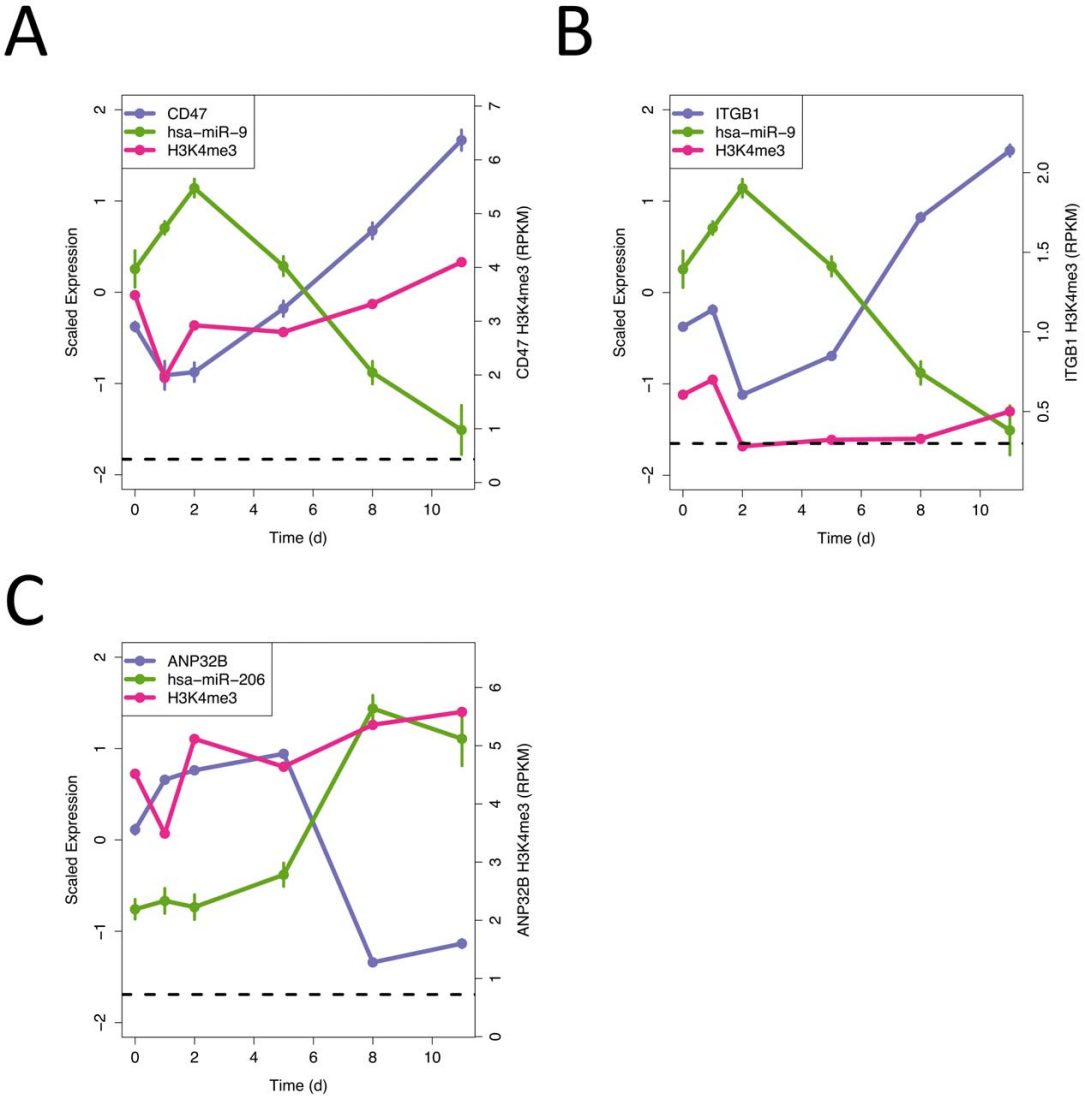
Epigenetic changes can be used to identify novel regulatory miRNAs. (A–C) Gene expression (blue), miRNA expression (green) and H3K4me^3^ levels for CD47, ITGB1 and ANP32B and the miRNAs associated with them. In all 3 cases the miRNA is predicted to regulate the relevant gene and is also anti-correlated in level. In (A) H3K4me^3^ levels correlate closely with the gene expression, whilst in (B & C) there is no correlation, suggesting a stronger role for the miRNA regulation.

Figure 4(A) shows the expression profile of CD47 and one of its predicted regulating miRNAs – miR-9. A clear anti-correlation is observed, as expected if miR-9 is regulating CD47 levels. miR-9 is known to be involved in insulin secretion [27], [40] as is CD47 and its receptor SHPS-1 [41]. However the existence of a functional link between the two has not been previously reported to our knowledge. A counter-argument to the importance of miR-9 on the regulation of CD47 expression is the H3K4me^3^ levels around the CD47 TSS. These levels correlate strongly with the gene expression implying that miR-9′s effect, if present, may only be small.

To avoid cases were H3K4me^3^ levels adequately explained the expression changes, next we turned our attention to those cases where the miRNA and gene expression levels were anti-correlated, but where the gene expression and H3K4me^3^ levels were poorly correlated. An example of this, again involving miR-9, is shown in Figure 4(B). The miR-9 target, Integrin Beta1 (ITGB1), has recently been shown to play a role in pancreatic development [42], but again the functional link between miR-9 and ITGB1 has not been reported previously. ITGB1 gene expression levels anti-correlate with miR-9 levels, but in this case the change in gene expression, particularly at later time points, cannot be well explained by changes in H3K4me^3^ which generally stays below the background threshold.

A final example where no clear links to pancreatic development exist for either the miRNA or the gene is shown in Figure 4(C). In this case the players are ANP32B, a histone chaperone and negative regulator for apoptosis [43], and miR-206, a miRNA known to be involved in myogenesis and to regulate the expression of other histone modifying genes [44], [45]. As with ITGB1, the H3K4me^3^ levels around the ANP32B TSS show little or no correlation with ANP32B expression levels, but strong anti-correlation with miR-206. This is particularly noticeable after day 8 where miR-206 expression suddenly jumps and ANP32B expression drops.

Since computational prediction of the regulatory effects of miRNAs remains a challenge, even after the integration of gene expression and epigenetic information as done here, experimental validation will be required to confirm functional roles in endocrine cell development for the miRNAs and miRNA-gene interactions we have identified.

### Canonical Signaling Pathways Involved in Development

The developmental and signaling pathways leading to endoderm specification have been well studied [46] and have served as the basis for this and other directed differentiation protocols. We therefore sought to validate our expression profiling system by first confirming that we could detect changes in the known signaling pathways being manipulated in this protocol. We used gene set enrichment methods to confirm the involvement of these pathways and to begin to search for novel regulators.

**Figure 5:**
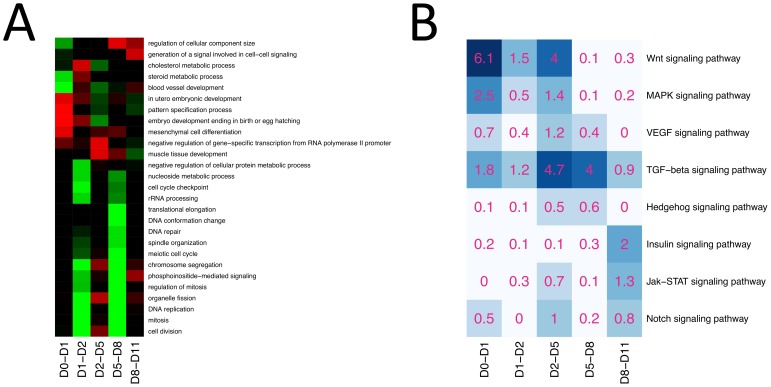
Functional analysis of gene expression changes at each time interval. (A) Heatmap of enrichment of GO terms amongst up and down-regulated genes at each time interval. Red indicates an enrichment amongst up-regulated genes and green an enrichment amongst down-regulated genes. (B) SPIA analysis of signaling pathways at each time point. The numbers in each cell indicate the -log10 of the P value for perturbation of the given pathway at each time interval. Darker colors indicate strong perturbations and white indicates no perturbation.

Figure 5(A) shows the result of a gene set enrichment analysis on the changes in gene expression at each time interval, using GO terms as the basis for the gene sets. Genes were called differentially expressed at each time interval based on a minimum fold change of 2 and multiple testing adjusted P value of less than 0.01. The number of differentially expressed genes was 718, 760, 1036, 1288 and 445 at day 0-day 1, day 1-day 2, day 2-day 5, day 5-day 8 and day 8-day 11 respectively. The most striking trend we observe in the gene set enrichment analysis is the sharp down regulation of genes involved in cell division that occurs in two waves: between day 1 and day 2 and between day 5 and day 8. Genes involved in phosphoinositide-mediated signaling (PI3K) follow a similar trend, which supports the previously described connection between PI3K signaling and endodermal differentiation [35].

Figure 5(B) shows a heatmap concentrating on intracellular signaling pathways as defined by KEGG [47]. The SPIA algorithm [48] was used to identify those pathways showing significant perturbations due to expression changes at each time interval. These correspond well with the known pathways and those manipulated through exogenous ligands. For example, during the first day of the differentiation (day 0-day 1) exogenous Wnt3a and Activin A are added to the culture media, and as expected, Wnt signaling shows large perturbations at this point. However, there is also a change in the Wnt signaling pathway from days 2–5, a period in which no exogenous Wnt ligand is added to the media, and therefore is likely driven by endogenously-produced Wnt signaling models. There is a robust induction of Wnt5a during this period of foregut patterning and expansion, consistent with mouse studies that indicate that Wnt5a regulates intestinal cell proliferation and gut expansion [49], [50] as well as studies that suggest a role for Wnt in pancreatic development [51].

**Figure 6:**
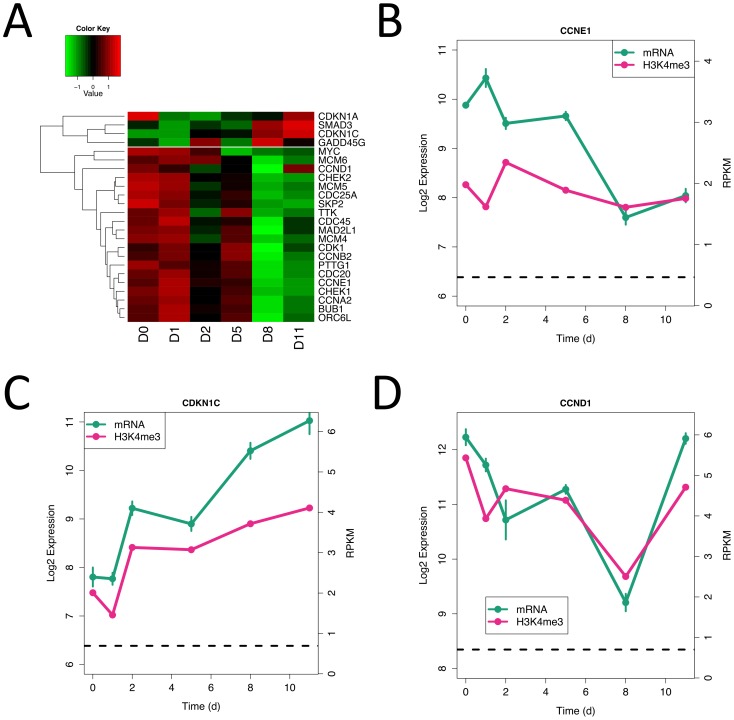
Expression of cell cycle genes occurs in bursts. (A) Heatmap of genes involved in cell cycle processes. (B–D) Gene expression (green) and H3K4me^3^ levels (red) for CCNE1, CDKN1C and CCND1.

Based on the results of the gene set enrichment analysis, we looked at cell cycle regulation in more detail. Figure 6(A) shows a heatmap of the expression levels of those cell cycle genes that change most significantly over the time-course. Most genes fall into the cluster exemplified by cyclin E1 (CCNE1) where expression generally falls with time, but particularly between days 1 and 2 and 5 and 8 (the time intervals identified above) (Figure 6(B)). The four genes not in this main cluster are cell cycle inhibitors such as CDKN1A/C and SMAD3. For these, gene expression increases over time (Figure 6(C)). These results suggest that cell proliferation decreases as the cells become more differentiated (as described above) and the involvement of SMAD3 suggests that this process may be driven by TGF-beta signaling [52]. An exception to the decrease in expression of cell cycle related genes at later time points is cyclin D1 (CCND1) whose levels closely match those of cyclin E1 until day 11, when its levels jump dramatically (Figure 6(D)). Levels of D1 and D2 cyclins have been shown to be essential for post-natal beta cell growth in mouse [53] and human models [54]. The beta cell precursors formed at day 11 seem then to be entering a cell-type specific proliferation regime regulated by these cyclins.

**Figure 7:**
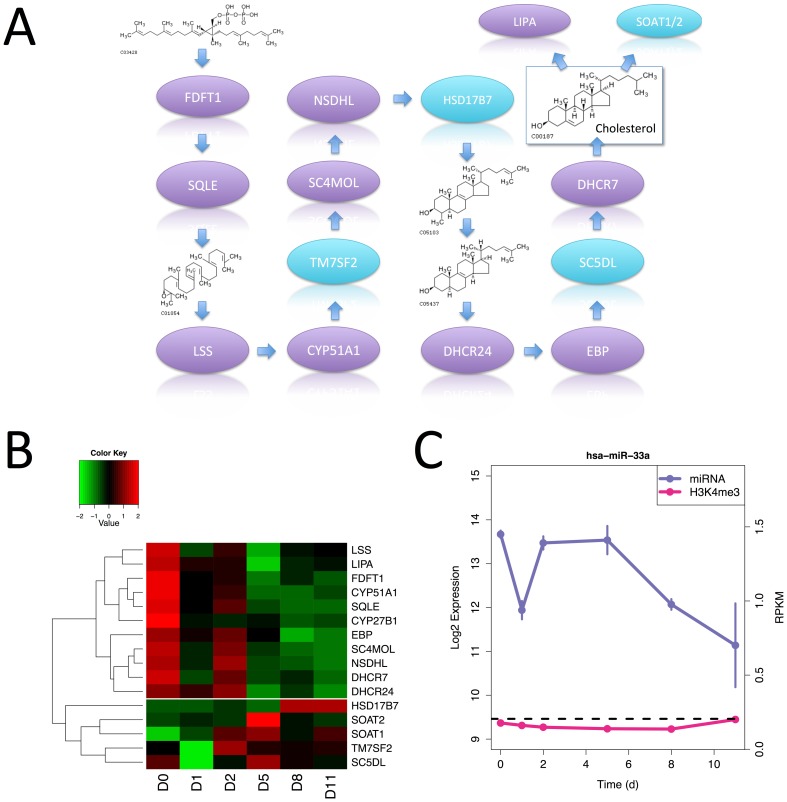
Expression of cholesterol metabolizing genes shows a strong time dependence. (A) The cholesterol biosynthesis pathway from squalene-PP (top left) to cholesterol (top right) showing all enzymes and some intermediates. Enzymes whose gene expression patterns fall into the largest cluster seen in the heatmap of the expression levels for these enzymes (B) are colored purple, other enzymes are light blue. LIPA and SOAT1/2 interconvert cholesterol and cholesterol ester. The cholesterol sensitive miRNA-33a shows a similar expression pattern (C) to the main cluster.

A novel observation from the gene set enrichment analysis is dramatic change in genes involved in cholesterol biosynthesis (Figure 7(A)) during early specification from pluripotent cell to foregut endoderm. Figure 7(B) shows a heatmap of the expression of the enzymes involved in cholesterol biosynthesis and Figure 7(C) shows the expression of miR-33, a miRNA linked to cholesterol homeostasis [55], [56]. Almost all of the genes encoding these enzymes show a sharp drop in expression at day 1 followed by recovery at day 2 and then a gentle decrease in expression. While the significance of this is unknown, cholesterol is, among other things, a precursor for steroid hormones such as estrogen and progesterone, and a recent report by Wong *et al*. links estrogen receptor signaling to embryonic stem cell proliferation and self-renewal [57]. Cholesterol is also known to play an important role in development, particularly through its role in the post-translational modification of the sonic hedgehog protein [58].

At the final time interval, insulin and Jak-STAT signaling become perturbed. Jak-STAT signaling has been linked to Ngn3 expression [59], but is most commonly linked to beta cell function in terms of the apoptotic response of cells to pro-inflammatory cytokines [60]. Having observed this perturbation at the signaling pathway level we returned to the gene level data and confirmed that expression levels of cytokines, such as IL-8, do increase dramatically at these time points (Figure S7). We suggest that the role of cytokine signaling, and Jak-STAT signaling in particular, may have an under-appreciated role in beta cell development and look at this in more detail in the next section.

### Causal Reasoning to Identify Novel Developmental Regulators

With the experimental system validated on the early stages of the differentiation, we then turned to the pancreatic induction stage (days 8–14) of the protocol where the complexity of cell types within the system increases dramatically. While the canonical signaling pathways involved in beta cell development are fairly well understood, not all of the individual regulators behind these processes had been fully identified by these pathway based approaches. We hypothesized that using our expression profiling data set, additional regulators of endocrine cell development could be identified using a novel network-based approach termed Causal Reasoning.

The Causal Reasoning Engine (CRE) is a method that utilizes manually curated, causal relationships. Each relationship encodes a conducted causal experiment in which a stimulus was applied to a biological system and the outcome recorded and published. Causal Reasoning was introduced in Pollard *et al.* [61] as a way to use this causal knowledge to predict causal drivers of observed large-scale transcriptional changes. We rely on a method published by Chindelevitch *et al.* [62] that introduced a statistically sound scoring scheme and showed that the method is reliable even when not all causal relationships are correctly specified. This last point is especially important as causal statements will originate from different biological contexts. Ultimately, Causal Reasoning provides a list of potential causal drivers (e.g. NEUROG3 up-regulation) with statistical quantification as to how many of the observed transcriptional changes are explained by it. Notably, these drivers are usually not implicated on a transcriptional level but rather refer to changes in abundance, activity or post-translational modifications of proteins.

We ran CRE on the gene expression changes between day 8 and day 11 to try and identify novel regulators of the process underlying endocrine precursor formation and endocrine cell development. Table 1 shows the top 20 protein hypotheses (potential causal drivers) ranked according to their Correctness score, i.e. the difference between the number of correctly and number of incorrectly explained transcripts. All hypotheses pass a p-value threshold of 10^−5^. A substantial proportion of these top-ranking hypotheses are well known to be involved in beta cell differentiation, proliferation and apoptosis. To demonstrate more formally that CRE is capable of correctly identifying causal drivers of beta cell function, we performed an enrichment analysis using results from literature mining. A genome-wide literature search shows that ∼4% of all human genes are associated with beta cell function in one or more published papers (see Materials and Methods for details of the literature analysis and synonyms used). In comparison, amongst the top 20 CRE hypotheses over 50% of genes are associated and amongst the top 50 hypotheses, ∼40% are associated with the same level of confidence. This is a highly statistically significant enrichment (hypergeometric P<1×10^−15^). We also observe that the proportion of drivers identified by CRE that are linked to beta cell function decreases as the lower ranked hypotheses are included (Figure S8), confirming that our approach is correctly identifying important causal drivers and that the ranking algorithm we use brings the most important drivers to the top of the list.

**Table 1:** Top 20 protein causal drivers of early pancreatic endoderm formation between day 8 and day 11.

Gene	Reg.	Corr.	Incorr.	Ambig.	Notes
**IL6**	Up	47	9	2	
**CDKN1A**	Down	39	7	2	−ve growth, +ve apoptosis
**THAP1**	Down	29	0	0	
**IL1B**	Up	48	20	5	−ve growth, +ve apoptosis
**NEUROG3**	Up	29	3	1	+ve differentiation
**EGF**	Up	29	9	3	+ve growth
**NCOR1**	Down	22	2	0	
**IL1A**	Up	35	16	2	
**TP53**	Down	36	18	2	+ve apoptosis
**HRAS**	Up	25	8	2	+ve apoptosis
**SOCS3**	Down	18	2	0	−ve growth
**TNFSF11**	Up	18	2	0	
**E2F1**	Up	18	2	1	+ve differentiation
**RET**	Up	21	6	0	
**SMAD3**	Up	17	5	0	−ve differentiation
**KDM5B**	Down	16	4	0	
**CXCR7**	Down	14	2	0	
**RB1**	Down	12	1	0	−ve growth
**TCF3**	Down	13	2	1	−ve apoptosis
**COL18A1**	Down	12	1	0	

The number of correctly, incorrectly and ambiguously explained gene expression observations are given for each gene as well as the predicted direction of regulation (up meaning activation/down meaning inhibition). The notes for each gene indicate that in cases where the gene is already associated with beta cell function whether it is generally considered a positive or negative regulator of beta cell differentiation, proliferation (growth) or apoptosis. All hypotheses pass correctness and enrichment p-value thresholds of 10^−5^.

**Figure 8:**
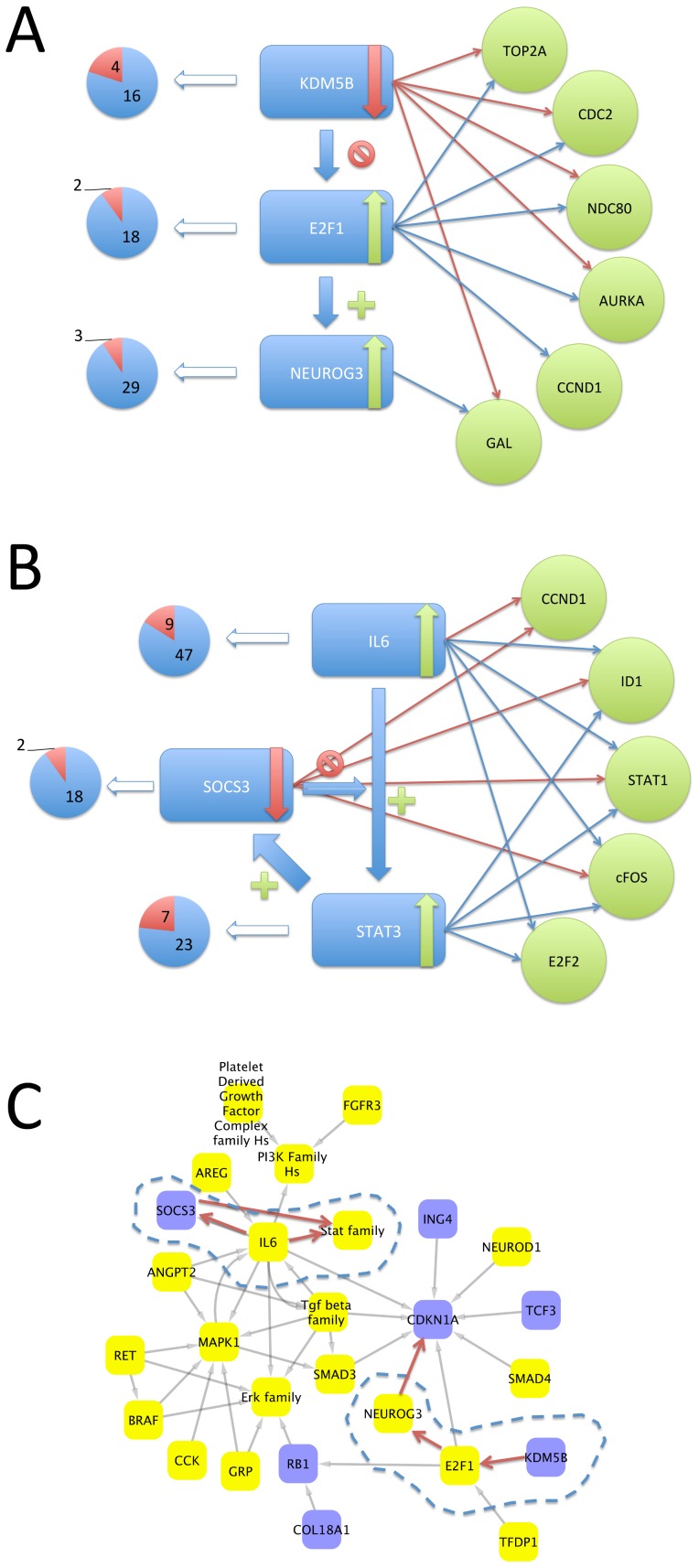
CRE identifies a number of novel pathways potentially involved in the final stages of endocrine pancreas development. (A) The KDM5B, E2F1 and NEUROG3 pathway described in the text. Arrows within the blue boxes indicate predicted increases (green) or decreases (red) in activity of the given protein between day 8 and day 11. The pie charts indicate the number of gene expression changes between day 8 and day 11 that are correctly (blue) and incorrectly (red) predicted. Genes regulated by each protein are indicated on the right. Blue arrows indicate activation of gene expression by the protein and red arrows indicate inhibition. The red stop symbol indicates that KDM5B is a known inhibitor of E2F1 expression and the green cross that E2F1 is a known activator of NEUROG3 expression. (B) The IL6, SOCS3 and STAT3 pathway. All details as for (A). (C) A network of causal drivers for gene expression changes between day 8 and day 11. Species predicted to have increased activity at day 11 are given in yellow boxes, species predicted to have decreased activity are given in purple. The two pathways discussed in the text are highlighted by dashed lines and red arrows. Grey arrows indicate known regulatory interactions between species (both activating and inhibiting).

Although the CRE hypotheses are enriched for genes known to be involved in beta cell function, there are also many hypotheses identified that have not previously been linked to this process. Combining these known and novel drivers allows us to build new pathways with potential roles in the regulation of this process. One such pathway can be built from the NEUROG3, E2F1 and KDM5B hypotheses. Taken together, these hypotheses correctly explain 8% of the total gene expression changes observed between day 8 and day 11. The number of correctly and incorrectly explained expression changes for each is shown in Figure 8(A). The role of NEUROG3 as a driver in beta cell function is well known [63], [64], but the roles of E2F1 and KDM5B are less well established.

Recent work has shown that E2F1 directly binds to and activates the NEUROG3 promoter in the embryonic pancreas [65] and ectopic E2F1 expression stimulates beta cell proliferation [66]. The causal analysis also highlights that E2F1 activity is known to increase CCND1 expression [67], which we have already shown is up-regulated at this time point, almost uniquely amongst cell-cycle regulators. We hypothesize that KDM5B’s role is even further up the pathway. Recent work has shown that KDM5B is a negative regulator of E2F1 in cancerous cell lines [68]. KDM5B and E2F1 share several downstream genes in common, as shown in Figure 8(A), mostly relating to cell cycle functions. There is also one gene in common between KDM5B and NEUROG3, which is the secreted hormone galanin. Based on these observations we would hypothesise that KDM5B may make a novel target for modulation of beta cell growth and differentiation *in vitro* and possibly *in vivo*.

The top ranked driver of gene expression from day 8 to day 11 was found to be IL-6. SOCS3, a downstream regulator of IL-6 signaling was also highly ranked, and this, along with the previous observation that cytokine driven Jak-STAT signaling appeared to be active at these later time points, pointed us towards a role for this pathway in beta cell function. SOCS3 has been linked with beta cell survival and proliferation previously [69], however the role of IL-6 appears to be less well appreciated. Although outside the top ranked hypotheses, STAT3, one of the principal transcription factors activated by IL-6, was also identified as a significant regulator. Together the IL-6, STAT3 and SOCS3 hypotheses explain 10% of the observed gene expression changes. The three components share several downstream genes, shown in Figure 8(B), including the beta cell specific cyclin CCND1 and ID1, STAT1 and cFOS.

Another potential novel driver of beta cell development identified by CRE is RET, the up-regulation of which is known to induce BRAF, hRAS and MAP kinase signaling. The RET hypothesis itself explains some 3% of the gene expression changes observed between day 8 and day 11. We were able to find one study linking RET to beta cell development [70], in which the authors show that GDNF, which signals through a complex involving RET, increases beta cell mass and proliferation. This pathway may make a novel *in vivo* target for modulation of beta cell regeneration.

RET feeds into a larger network we derive from the top ranked causal drivers and shown in Figure 8(C). This network shows the largest connected component of the network of causal drivers with P<1×10^−4^, including protein nodes and protein families. The network involves factors well known to be involved in beta cell function, such as NEUROG3, NEUROD1 [71], [72] and CDKN1A, but also a number of novel regulators. CDKN1A forms one of the most notable hubs in this small network and has links feeding into it from both the IL-6 and KDM5B/E2F1/NEUROG3 pathways identified above. As has been proposed recently, modulation of this central cell cycle inhibiting regulator may be necessary to allow beta cell proliferation *in vivo* [73].

### Experimental Validation of CRE Predictions

We then went back to the cell differentiation protocol to evaluate predictions from the CRE, using the top hypothesis IL-6 as our test case. The CRE analysis predicted that signaling through the IL-6 pathway will positively affect NEUROG3 expression, and thus promote the formation of new endocrine cells. We were able to detect expression of the gene for the IL6 receptor (IL6R) in all of the day 11 replicates (P<0.05). We tested this hypothesis by adding exogenous IL-6 to the culture media and looked for gene signatures indicative of new endocrine cell formation. Recombinant IL-6 (100 ng/ml) was added during stage 4 (days 8–14) of the differentiation and gene expression changes analyzed by qRT-PCR at day 14. To facilitate the detection of IL-6 mediated changes, Noggin, a powerful inducer of NEUROG3 expression, was omitted from the culture media during the IL-6 treatments.

**Figure 9:**
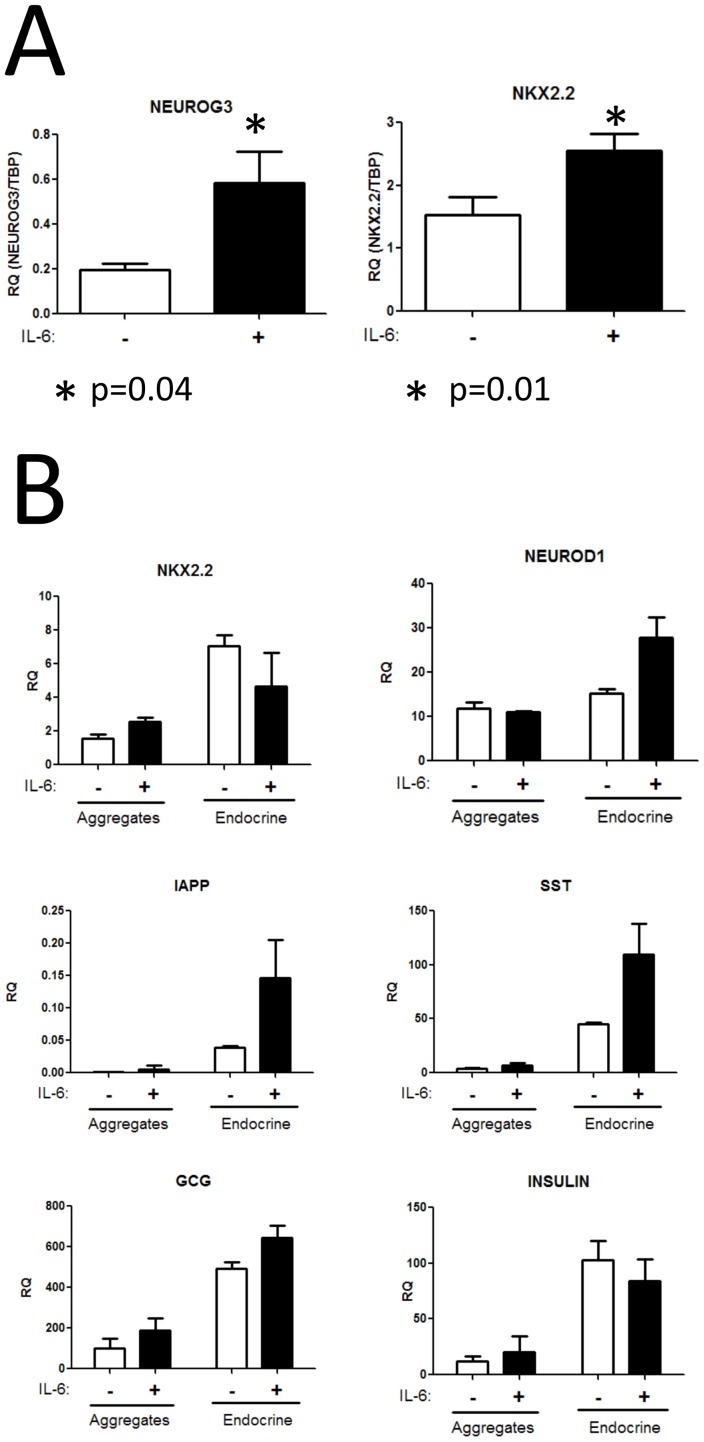
IL6, the top CRE prediction, has effects on expression of endocrine markers. (A) Treatment of pancreatic aggregates with IL-6 induces de novo gene expression of the pro-endocrine transcription factors NEUROG3 and NKX2.2, indicating commitment of pancreatic progenitor cells into the endocrine lineage. Noggin induction of these genes resulted in 8-fold increases (data not shown) (B) Gene expression in response to IL-6 was compared between whole aggregates (mixture of pancreatic progenitors and endocrine cells) and cultures of enriched endocrine cells (depleted of pancreatic progenitors). Induction of NKX2.2 expression was only seen in whole aggregates, consistent with the role of IL-6 in converting pancreatic progenitors into new endocrine cells. Enhanced expression of NEUROD1, IAPP, and SOMATOSTATIN seen in response to IL-6 in purified endocrine cells, suggesting IL-6 has additional roles in committed endocrine cells. No significant differences seen in INSULIN or GCG gene expression. Statistical testing using a standard t-test was performed.

As predicted by the CRE analysis, addition of IL-6 significantly up-regulated mRNA expression of the endocrine progenitor gene NEUROG3 by approximately 3-fold (Figure 9(A)). NKX2.2, which is genetically downstream of NEUROG3 and a marker of committed endocrine cells, was also significantly induced, indicating that IL-6 was sufficient to drive the endocrine program and suggests an increase in the number progenitor cells differentiating into endocrine cells.

However because the aggregates in these cultures are a mixture of both pancreatic progenitor cells and newly differentiated endocrine cells (Figure S1), it is impossible to know which cell type is responding to IL-6 to induce NKX2.2 expression. This enhanced expression could either represent an increase in the total number of cells in the culture that have become endocrine and are now expressing NKX2.2, or alternatively it could simply reflect an increase in expression within cells that are currently expressing the gene. To address this question, we took advantage of the inherent physical differences between pancreatic epithelial cells and their endocrine cell progeny.

Pancreatic progenitor cells exist as tightly connected cords of epithelial cells that require extensive cell-cell contact for survival and function, and disruption of these contacts leads to apoptosis/anoikis. In contrast, immature pancreatic endocrine cells are by nature migratory and require minimal cell-cell contact, and are thus refractile to anoikis. By enzymatically dissociating our culture into single cells and placing these cells back into culture, the progenitor cells die off and the remaining endocrine cells will survive and self-organize into spheroid clusters. Using this method, we can achieve enrichment of the endocrine cells to >90% purity (Figure S9), and can thus address the effect of IL-6 on the endocrine cells with minimal mRNA contribution from the pancreatic progenitor fraction.

As described above, stage 4 pancreatic cells were dissociated and allowed to spontaneously re-form into islet like endocrine clusters. The following day, these endocrine clusters were treated with either DMSO or IL-6 and gene expression analyzed 48 hours later (Figure 9(B)). Intact aggregates containing both pancreatic progenitors and endocrine cells were treated in parallel as a control.

As seen in the previous experiment, addition of IL-6 to the mixed culture of pancreatic progenitors and endocrine cells (aggregates) induced NKX2.2 expression, however no change was seen in the purified endocrine cells. This supports the hypothesis that IL-6 is acting on pancreatic progenitor cells to induce new endocrine cell formation. Interestingly, changes in the expression of a number of additional genes were detected in purified endocrine population, including the secreted hormones IAPP and somatostatin (though not insulin), supporting the previously reported effect of IL-6 on islet cell function [74], and indicating that IL-6 may play multiple roles in the specification and maturation of embryonic endocrine cells.

## Discussion

The aim of this study was to better understand and predict the pathways involved in endocrine development and use a novel causal reasoning approach to identify new opportunities toward the development of efficient islet cell replacement therapy. Due to the limited supply of available human islet cells, the production of pancreatic endocrine cells from an alternative source would be required for efficient cellular replacement therapy. Currently, several groups have reported generating these cells from human embryonic stem cells, although the true functionality of the cells created by many of these protocols remains open to question [75]. The most successful of these methods succeeded in creating immature human pancreatic endoderm that, when transplanted into a rodent diabetes disease model, matured into functional hormone-producing endocrine tissue and fully restored the normal glycemic state of a diabetic animal [4]. This process, however, takes more than 8 weeks and is not well defined due to the maturation occurring *in vivo*. Using this same model system as a platform, we sought to further understand the gene expression changes that contribute to pancreatic endocrine cell formation. The high cell purity, coupled with the synchronous response of these cells to the inductive cues provided in the protocol make it particularly amenable to detailed transcriptome analysis at each stage from pluripotent cell through the early endoderm and foregut stages.

We showed that this protocol is highly reproducible and can produce pancreatic endocrine precursor cells that show appropriate gene expression. Integration of epigenetic data showed that changes in H3K4me^3^ levels can account for a large percentage of the gene expression changes observed, though clearly many other such histone and direct DNA marks will also have important roles to play. Controlled modulation of the enzymes responsible for the addition and removal of such marks along with the targeting machinery that lead them to their correct sites of action in the genome is likely to be a key research goal for directed differentiation studies in many therapeutic areas. We identify one histone demethylase, KDM5B, as a potential target for modulation in the context of endocrine beta cell production due to its modulation of E2F1 and NEUROG3.

The complexities of biological regulation at the epigenetic and transcriptional levels are made clear by Figure 2. Unlike the majority of genes, the expression of insulin, as well as other endocrine hormones, appears not to be regulated epigenetically, at least in terms of H3K4me^3^ levels. Whether this is an artifact of the precursor nature of these cells is not clear from our *in vitro* data, but a similar observation was made recently using islet cells [14]. Clarification of this will require the determination of the precise chromatin structure around these genes in precursors as well as mature beta cells.

The role of canonical signaling pathways in beta cell development has been well studied and, as shown in Figure 3, our analysis agrees with the consensus opinion of the importance, timing and role of these pathways. The two exceptions to this are Jak-STAT signaling and cholesterol mediated signaling. In the latter case, the evidence for a role comes from apparently highly coordinated changes in the levels of cholesterol producing enzymes as well as other genes involved in cholesterol homeostasis, such as the miRNA mir-33. The obvious point for cholesterol to feed into the developmental decision making machinery is via Hedgehog signaling as it is a known post-translational modification of the Sonic Hedgehog protein. Further investigation of the links between cholesterol metabolism, Hedgehog signaling and pancreatic development seem warranted.

The importance of JAK-STAT signaling in the later stages of the protocol, particularly via IL-6, was also detected by our causal reasoning approach. Causal reasoning is beginning to be recognised as an important tool within the field of drug discovery. Another study has used this approach to identify common mechanisms activated within drug-treated cell lines in an oncology-related programme [76], which highlights the generation of similar types of biological networks to those, described here. Here we relied on a specific implementation of the approach that provides an accurate statistical quantification of significance for predicted upstream drivers.

Whilst the nature of the causal reasoning analysis means that no single link can ever be truly novel, bringing several links together can reveal novel pathways. For instance, the links between the histone demethylase KDM5B and the transcription factors E2F1 and NEUROG3 have only recently been established in isolation. Our analysis brings them together in a new light and suggests an important new pathway for the regulation of beta cell proliferation.

We then tested our CRE approach using the top-ranked prediction of IL-6 as a positive regulator of endocrine cell formation. We showed that addition of exogenous IL-6 to our pancreatic precursor population resulted in a significant increase in NEUROG3 and NKX2.2 expression in these cells. Interestingly, upon separating out the precursor population and a purified endocrine population our results demonstrated that the effect was only observed in the pancreatic precursors indicating that more cells have become committed to the endocrine lineage. Further experiments will be required to fully validate the CRE based predictions as well as the other observations made relating to miRNA and epigenetic regulation of differentiation.

In conclusion, we have uncovered novel pancreatic endocrine maturation pathways based on genomic profiling and pathway analysis. In particular, the causal reasoning approach in conjunction with more traditional pathway analysis methods seems to be a promising emerging tool to point at concise molecular hypotheses driving observed transcriptional changes and lends itself well to direct experimental follow-up. Beyond the several well-known drivers of pancreatic differentiation and the now validated IL-6 hypotheses, the approach predicted a number of other strong drivers. These hypotheses should be investigated further in future studies to get a more concise picture of the pathways involved in pancreatic cell differentiation.

## Materials and Methods

### Cell Culture Methods

CyT49 (licensed from Viacyte Inc., proprietary male hESC line, normal karyotype) was grown as a monolayer culture by their standard methods [K. D’Amour, personal communication]. Briefly, cells were thawed at 10×10∧6 cells per T175 flask and grown in DMEM/F12 media (Gibco), 10% Xenofree Knockout Serum Replacement (Gibco), 1× non-essential amino acids (Gibco), 1% Glutamax (Gibco), 1% penicillin/streptomycin plus 10 ng/ml activin A and Heregulin β1 at 37°C and 8% CO_2_. Prior to initiating cellular differentiation, formation of cellular aggregates was achieved by placing the single cell suspension of undifferentiated ESCs into a rotational culture in 6-well, low attachment tissue culture plates in ESC growth media. Plates were rotated at 95 rpm overnight. The differentiation was also performed in rotating suspension culture and plates were rotated at 95 rpm days 0–3 and 11–12 and 110 rpm days 4–10 at 37°C and 8% CO_2._ For initiation of differentiation (as described in [23]), the aggregates were pooled and washed with PBS prior to resuspending in day 0 differentiation media of RPMI, ITS (1∶1000), activin A (100 ng/ml) and Wnt3a (50 ng/ml) Thereafter, media was changed daily according to the schedule (day 1– RPMI with 0.2% vol/vol FBS, ITS (1∶1000) and activin A (100 ng/ml), day 2– RPMI with 0.2% FBS, ITS (1∶1000), KGF (25 ng/ml) and TBF inhibitor IV (2.5 µM), day 3 - RPMI with 0.2% FBS, ITS (1∶1000) and KGF (25 ng/ml), day 5– DMEM (HiGlucose) with 1% B27 supplement, KAAD-cyclopamine (0.25 µM), TTNPB (3 nM), KGF (50 mg/ml), EGF (50 ng/ml) and noggin (50 ng/ml), day 8– DMEM (HiGlucose) with 1% B27 supplement, KGF (50 mg/ml), EGF (50 ng/ml) and noggin (50 ng/ml) by gentle aspiration from the well of ¾ media volume and replacement. Cells were removed for Illumina microarrays on days described in the text (5×10^6^ cells for d0 time-point or 2000 aggregates for day 1–11) and stored as frozen cell pellets. RNA was prepared using Qiagen RNAeasy kit according to manufacturer’s instructions. 10×10∧6 day 0 cells and 3000 aggregates (day 1–11) were fixed in 11% formaldehyde for ChIP-seq as described http://www.genpathway.com/support/fixation.html.

Endocrine purification - On day 13, PE aggregates were pelleted into a 50 ml conical tube, the supernatant was aspirated, 15 ml pre-warmed accutase added to the tube, and incubated at 37°C until fully dissociated. The single cells suspension was transferred to another tube and quenched with 25 ml of media. Cells were spun@1000 rpm for 5 min and resuspended in 10 ml of db-K50E50N50 media. Approximately 6 million cells were added in 5.5 ml media to each well in a low-binding 6 well plate on a shaker @95 rpm and allowed to spontaneously re-aggregate.

### Intraperitoneal Glucose Tolerance Test in SCID-bg Mice

All animal procedures were approved by the Pfizer Institutional Animal Care and Use Committee. Eight weeks post hESC-derived progenitor cell implantation, SCID-bg mice were fasted overnight. Starting at ∼9∶00 AM, basal glucose values were recorded and a basal blood sample was obtained for human C-peptide analysis. Mice were then dosed i.p. with a 30% glucose solution at 10 ml/kg for a 3 g/kg glucose load. At 60 minutes post-glucose load glucose values were recorded and a second blood sample was obtained for human C-peptide analysis. Blood samples were collected into serum separator tubes, spun at 4°C and serum was assayed the same day for human C-peptide using the Mercodia Ultrasensitive, human selective, C-peptide ELISA.

### Microarray Hybridization and HTS-Seq Methods

The Illumina analysis was carried out by GeneLogic according to manufacturer’s instructions. ChIP-seq was carried out by GenPathway utilising a proprietary immuno-precipitation method with the H3K4me^3^ antibody (ab6000) and their HistonePath technology.

### Data Normalization and Analysis

The mRNA, miRNA and ChIP-Seq profiling data sets are available through ArrayExpress (accessions: E-MTAB-817, E-MTAB-818, E-MTAB-821 respectively). Normalised datasets are also available in supplementary tables 4–6. All microarray data was log. base 2 transformed and quantile normalized using the beadarray [77] package in Bioconductor [78]. Probes detected in less than 3 samples (Detection P<0.01) were excluded. H3K4me^3^ ChIP-Seq reads were aligned to the hg18 reference human genome with Eland and processed using the ShortRead [79] package. Reads were filtered by base and alignment quality. Coverage was calculated in 5 kbp windows around TSSs defined by Ensembl [80] and normalized to reads per kilobase per million (RPKM). MAC [32] was used to detect H3K4me^3^ ChIP peaks at each time point relative to a control sample using the same parameters as Stitzel *et al* [14].

Differential gene expression was performed using limma. Criteria for differential expression were as described in the main text. Gene set analysis was performed using gene sets defined by GO [81] and either the limma or GOstats packages. SPIA [48] analysis was performed on KEGG [47] signaling pathways.

### Causal Reasoning Engine Algorithm

The Causal Reasoning Engine (CRE) follows the general data model introduced in Pollard *et al*. [61]. The CRE algorithm used in this article was introduced by Chindelevitch *et al*. [82] and provides novel statistical measures to assess relevance of uncovered upstream regulators to plausibly interpret the observed expression changes. Briefly, the approach relies on a large collection of curated causal statements of the form:

**A** [increases or decreases] **B**, where **A** and **B** are measurable biological entities.

The biological entities can be of different types (e.g. phosphorylated proteins, transcript levels, biological process and compound exposure) and each statement is tied to accessible, peer-reviewed articles. For this work, we licensed approximately 450,000 causal statements from commercial sources (Ingenuity Systems and Selventa).

Each biological entity in the network and its assumed mode of regulation is a potential *hypothesis (e.g. predicted decrease in PPARG transcription activity)*. For each hypothesis, we can now compare all possible downstream transcriptional changes in the knowledge base with the observed transcriptional changes in the experiment. We consider two metrics to quantify the significance of a hypothesis with respect to our experimental data set, namely enrichment and correctness. The *Enrichment* p-value for a hypothesis h quantifies the statistical significance of finding *(#incorrect+#correct)* transcripts within the set of all transcripts downstream of h. The exact p-value can be computed by a Fisher’s exact test. This is a standard approach in gene set enrichment methods and does not take the direction of regulation into account [83].

The *Correctness* p-value is a measure of significance for the score of a hypothesis h defined as (*#correct – #incorrect*). As desired, this score is high, if the number of correct prediction exceeds the number of incorrect predictions. To ensure statistical significance under a null model of randomly re-assigning up- and down-regulated transcripts to arbitrary nodes, we compute the distributions for this score and derive appropriate p-values. Surprisingly, the distributions can be computed analytically in polynomial time using combinatorial programming approaches.

The Causal Reasoning Engine is implemented in the statistical programming language R and uses the igraph package for representation of the network of causal assertions.

### Literature Analysis

To assess prior literature associations of top CRE hypotheses, we applied an internally developed text-mining algorithm that detects relevant co-occurrence patterns between protein and gene mentions and other biomedical concepts of interest (e.g. diseases, cell lines) in Medline abstracts. Briefly, the method detects entity occurrences based on synonym dictionaries. It assesses relevance of a co-occurrence pattern using a rule-based approach. Rules indicating relevance of a particular co-occurrence include the number of mentions of each entity or its synonyms in the text, whether the entities are mentioned in the title as well as the proximity of occurrence, e.g. within one sentence (Roberts *et al*., manuscript in preparation).

We applied this method to detect relevant co-occurrences in all Medline abstracts as of January 2011. We focused on the ∼7800 protein entries used in the CRE method and present in our internal dictionary co-occurring with the term “pancreatic beta cells” (synonyms used: pancreatic beta cell, pancreatic beta cells, islet-beta cells, islet-beta cell, beta cell differentiation, pancreatic differentiation, beta-cell regeneration, insulin-expressing cell, insulin-expressing cells, islet cell formation, islet cell differentiation, mature islets, mature islet cells).

## Supporting Information

Figure S1 **(A) Cell composition at selected stages of differentiation.** Cell aggregates were dissociated into single cells and analyzed by fluorescent activated cell counting. Aggregates at day 0, prior to the initiation of differentiation, were uniformly OCT-4 positive. As the cells progress through the differentiation into to definitive endoderm and through the foregut endoderm stage, >95% of the culture expresses CXCR4 (day 2) and FOXA2 (day 5). Transition through these stages was also confirmed by qRT-PCR (data not shown). By the end of the differentiation at stage 5, the majority of the culture consists of either endocrine cells (Chromogranin A-positive), or pancreatic progenitors (PDX1/NKX6.1 co-positive, ChromograninA-negative). A variable proportion of the culture (between 2–20%) will consist of cells outside of these two primary cell fates but still restricted to the endoderm/foregut lineage. (B) Cell diversity at end of Stage 4. At the end of the 14 day differentiation procedure, the culture consists of a variety of cell lineages. FACS analysis of the bulk culture demonstrates that approximately 50% of the express the pan-endocrine marker ChromograninA. Analysis of cells expressing insulin and glucagon within this ChromograninA population demonstrates a variety of endocrine subtypes, with approximately 13% of the cells in the ChromograninA population that co-express insulin and glucagon. Gene expression analysis of these day 14 cultures reveals the expression of the other endocrine hormones (log scale). Low off target differentiation into intestine (CDX2) liver (ALBUMIN), anterior endoderm (FOXE1) or mesoderm (MEOX1) can also be seen by RNA analysis of the day 14 cultures. (C*) In vivo* function of implanted pancreatic progenitors. Cell aggregates used in the in vitro gene expression analysis were allowed to differentiate *in vivo* and were subjected to glucose tolerance tests (3 g/kg i.p.) at 8 weeks and 6 months post-implant. By 8 weeks basal human C-peptide was detectable, with significant stimulation in response to glucose challenge. By 16 week post-implant, implanted cell grafts will typically secrete human C-peptide greater than 2,000 pM and are considered functionally mature. As can be seen in this cohort at 6 months, the implanted grafts secreted greater than 3,000 pM C-Peptide by 30 minutes post-glucose challenge.
(PDF)

Figure S2 **(A) Genome wide miRNA expression correlation heatmap between samples.** Samples are clustered by the Euclidean distance between rows/columns and single linkage clustering. The colored bar along the top of the heatmap indicates the timepoint at which the sample was taken (pink: day 0, maroon: day 11). (B) Genome wide H3K4me^3^ level correlation heatmap between samples. All details as (A).
(PDF)

Figure S3 **Plot of each sample on the first two components from principal component analysis (PCA) based on gene expression (A), miRNA expression (B) and H3K4me^3^ levels (C).**
(PDF)

Figure S4 **Plot of gene expression data as measured by array (green) and qPCR (orange) for NANOG (A), POU5F1 (B), NKX2-2 (C) and SOX17 (D).** All data is normalized such that D0 expression equals 1.
(PDF)

Figure S5 **Histogram of the correlation coefficients (R) for each gene between expression levels measured by arrays and qPCR.**
(PDF)

Figure S6 **Plot of H3K4me^3^ read density around transcriptional start sites (TSS) as defined using Ensembl.**
(PDF)

Figure S7 **Gene expression (blue) and H3K4me^3^ levels (red) at each timepoint for IL-8.**
(PDF)

Figure S8 **The proportion of CRE hypotheses linked in the literature to beta cell development as a function of the rank cutoff. Genome wide the proportion is ∼5%.** FACS of purified endocrine cell population compared to PE aggregates.
(PDF)

Figure S9 **FACS of purified endocrine cell population compared to PE aggregates.**
(PDF)

Table S1 **List of 113 genes tested by qRT-PCR.** All primer pairs used were human specific except where denoted M for murine specific.
(PDF)

Table S2 **Top 20 differentially expressed genes: List of the 20 genes showing the most significant expression changes at each time interval (D0–D1, D1–D2, D2–D5, D5–D8 and D8–D11).** Illumina probe and target IDs are given along with the log base 2 fold change (‘logFC’), t statistic (‘t’), raw P value (‘P.Value’) and Benjamini-Hochberg corrected P value (‘adj.P.Val’). All other columns are standard output from limma.
(XLS)

Table S3 **Top 20 differentially expressed miRNAs: List of the 20 miRNAs showing the most significant expression changes at each time interval (D0–D1, D1–D2, D2–D5, D5–D8 and D8–D11).** Illumina probe IDs are given along with the log base 2 fold change (‘logFC’), t statistic (‘t’), raw P value (‘P.Value’) and Benjamini-Hochberg corrected P value (‘adj.P.Val’). All other columns are standard output from limma.
(XLS)

Table S4 **Normalised data for mRNA: The full mRNA expression dataset giving expression levels for the indicated Illumina probes after log2 transformation and quantile normalization as described in the Materials and Methods.** Column headers indicate the day (‘D0’), biological replicate (A–C) and technical replicate (1/2).
(TXT)

Table S5 **Normalised data for miRNA: The full miRNA expression dataset giving expression levels for the indicated Illumina probes after log2 transformation and quantile normalization as described in the Materials and Methods.** Column headers indicate the day (‘D0’), biological replicate (A–C) and technical replicate (1/2).
(TXT)

Table S6 **Normalised data for ChIP-seq: Total H3K4me^3^ ChIP-Seq read counts within 5** **kbp of the TSS of each human gene for each time point.** The read count for the control sample (without H3K4me^3^ antibody) is given as ‘CTRL’.
(TXT)
