# Supplementary figures and images for: Novel Pancreatic Endocrine Maturation Pathways Identified by Genomic Profiling and Causal Reasoning
Source: PLoS One. 2013 Feb 13;8(2):e56024. doi: 10.1371/journal.pone.0056024 (PMC3572136; doi:10.1371/journal.pone.0056024)

**A**

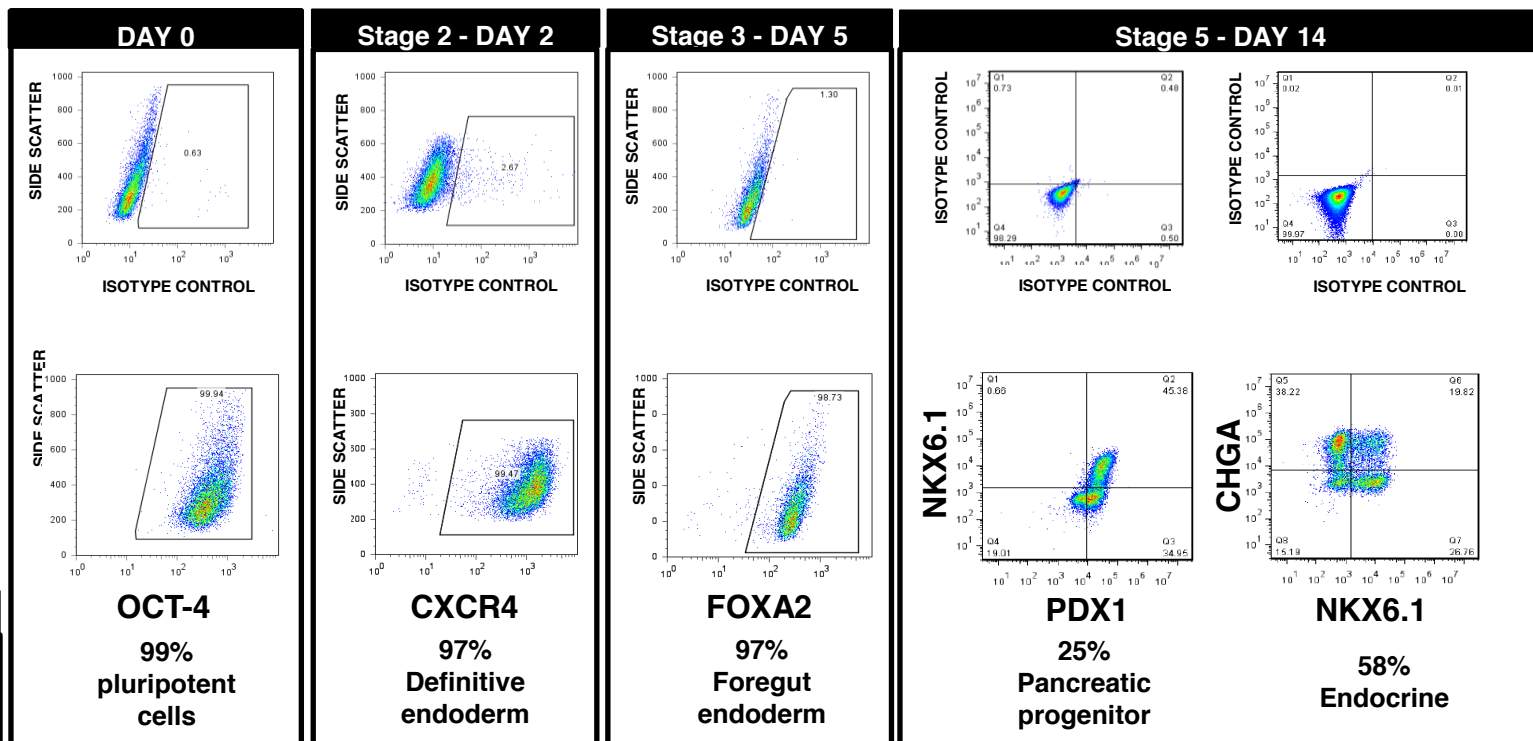

**B**

**Stage 5- DAY 14**

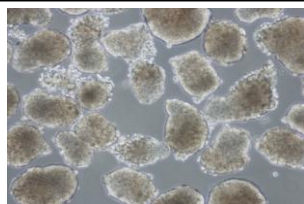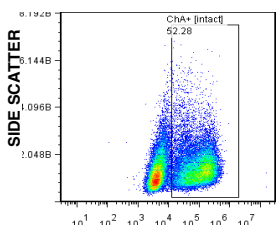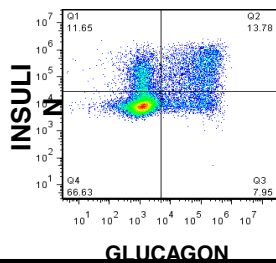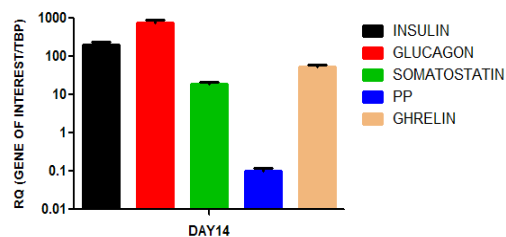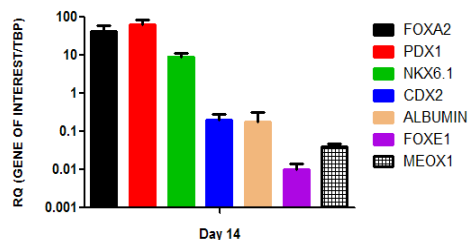

**C**

**Human C-Peptide**

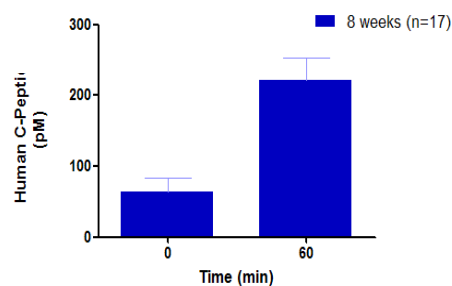

**Human C-Peptide**

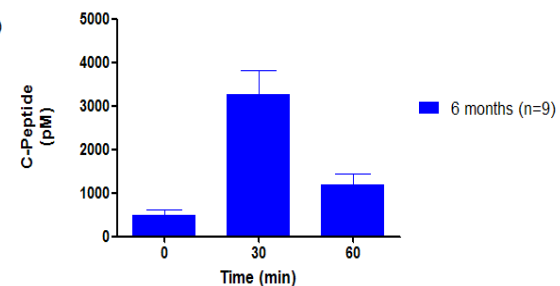

Figure S1\_Gutteridge *et al.* 2012

Supplement: Figure S1 — (A) Cell composition at selected stages of differentiation. Cell aggregates were dissociated into single cells and analyzed by fluorescent activated cell counting. Aggregates at day 0, prior to the initiation of differentiation, were uniformly OCT-4 positive. As the cells progress through the differentiation into to definitive endoderm and through the foregut endoderm stage, >95% of the culture expresses CXCR4 (day 2) and FOXA2 (day 5). Transition through these stages was also confirmed by qRT-PCR (data not shown). By the end of the differentiation at stage 5, the majority of the culture consists of either endocrine cells (Chromogranin A-positive), or pancreatic progenitors (PDX1/NKX6.1 co-positive, ChromograninA-negative). A variable proportion of the culture (between 2–20%) will consist of cells outside of these two primary cell fates but still restricted to the endoderm/foregut lineage. (B) Cell diversity at end of Stage 4. At the end of the 14 day differentiation procedure, the culture consists of a variety of cell lineages. FACS analysis of the bulk culture demonstrates that approximately 50% of the express the pan-endocrine marker ChromograninA. Analysis of cells expressing insulin and glucagon within this ChromograninA population demonstrates a variety of endocrine subtypes, with approximately 13% of the cells in the ChromograninA population that co-express insulin and glucagon. Gene expression analysis of these day 14 cultures reveals the expression of the other endocrine hormones (log scale). Low off target differentiation into intestine (CDX2) liver (ALBUMIN), anterior endoderm (FOXE1) or mesoderm (MEOX1) can also be seen by RNA analysis of the day 14 cultures. (C) In vivo function of implanted pancreatic progenitors. Cell aggregates used in the in vitro gene expression analysis were allowed to differentiate in vivo and were subjected to glucose tolerance tests (3 g/kg i.p.) at 8 weeks and 6 months post-implant. By 8 weeks basal human C-peptide was detecta [file pone.0056024.s001.pdf]

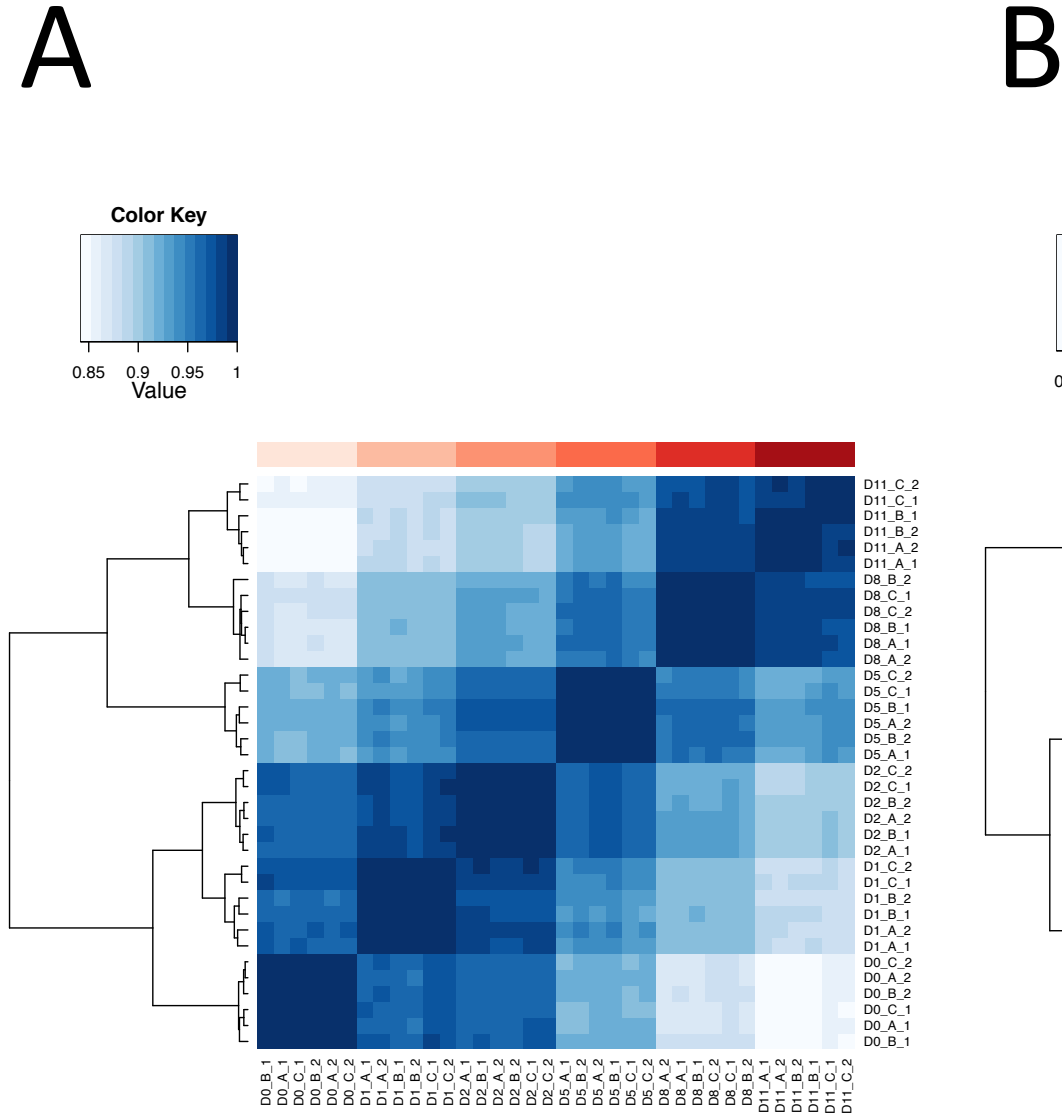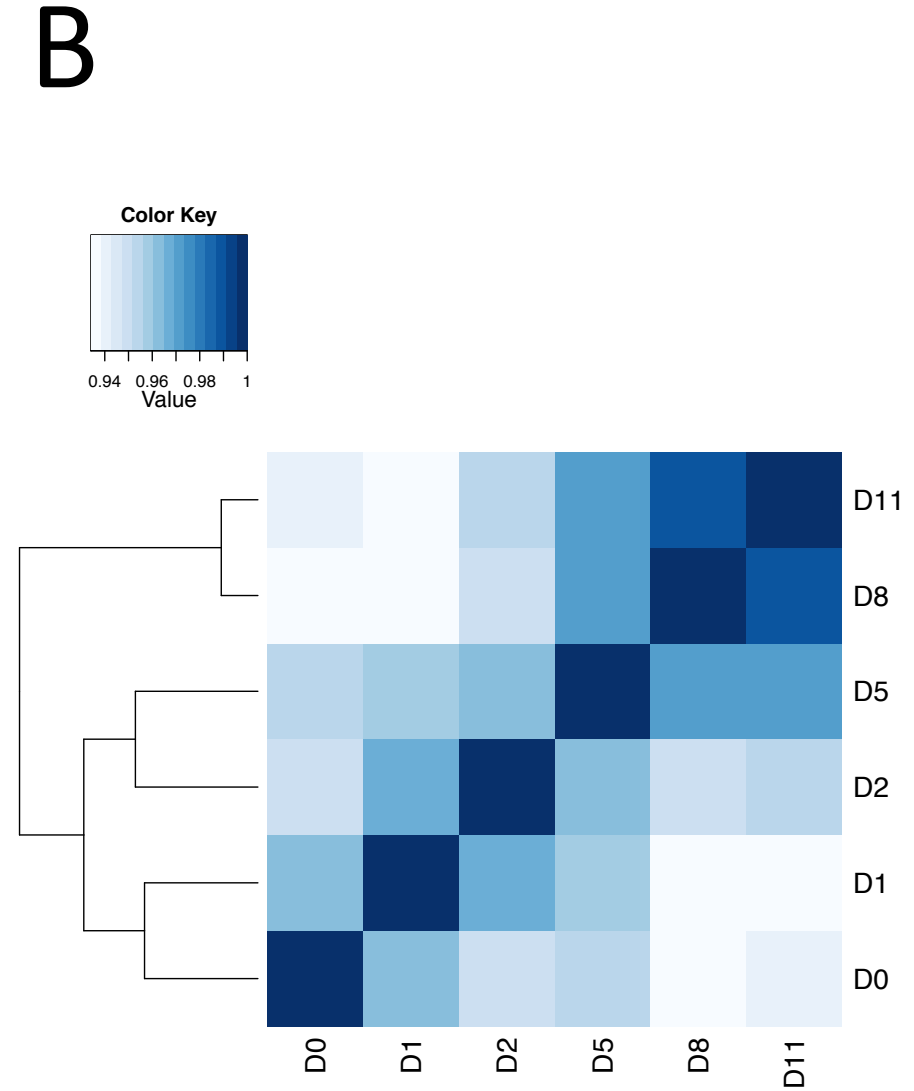

Supplement: Figure S2 — (A) Genome wide miRNA expression correlation heatmap between samples. Samples are clustered by the Euclidean distance between rows/columns and single linkage clustering. The colored bar along the top of the heatmap indicates the timepoint at which the sample was taken (pink: day 0, maroon: day 11). (B) Genome wide H3K4me3 level correlation heatmap between samples. All details as (A). (PDF) [file pone.0056024.s002.pdf]

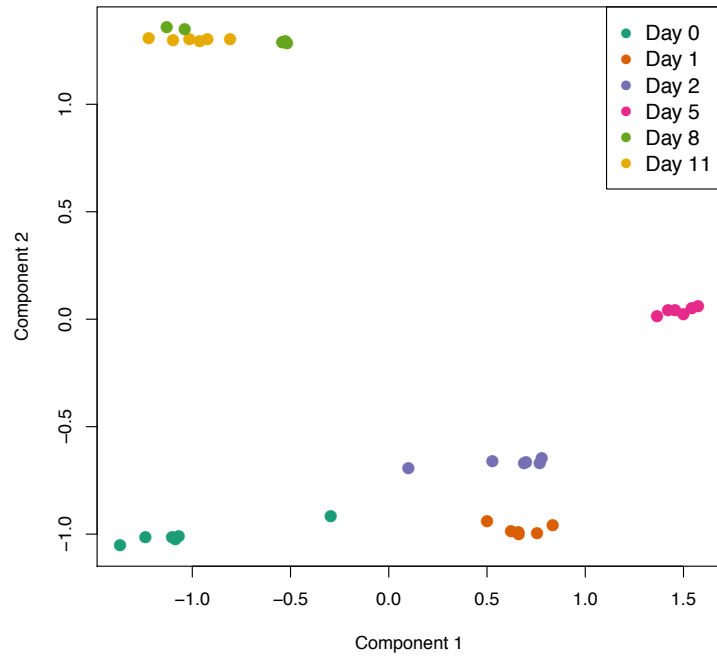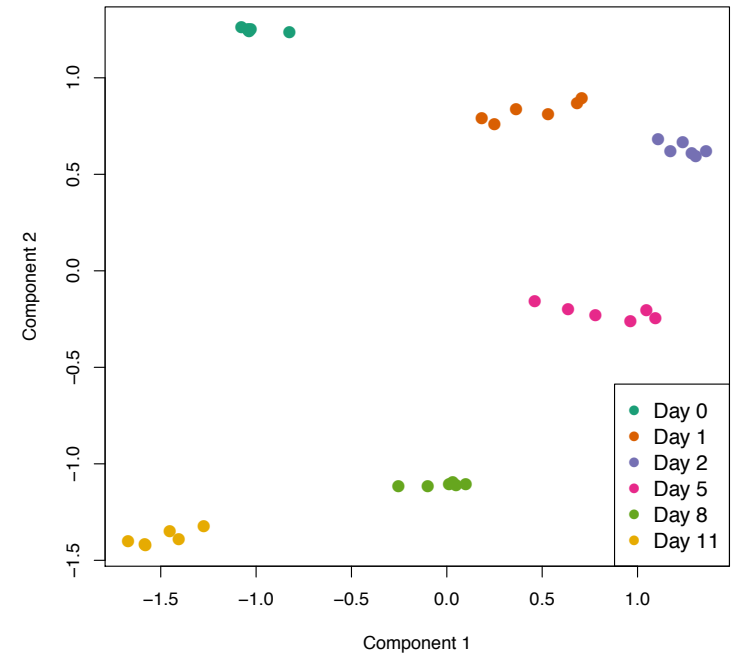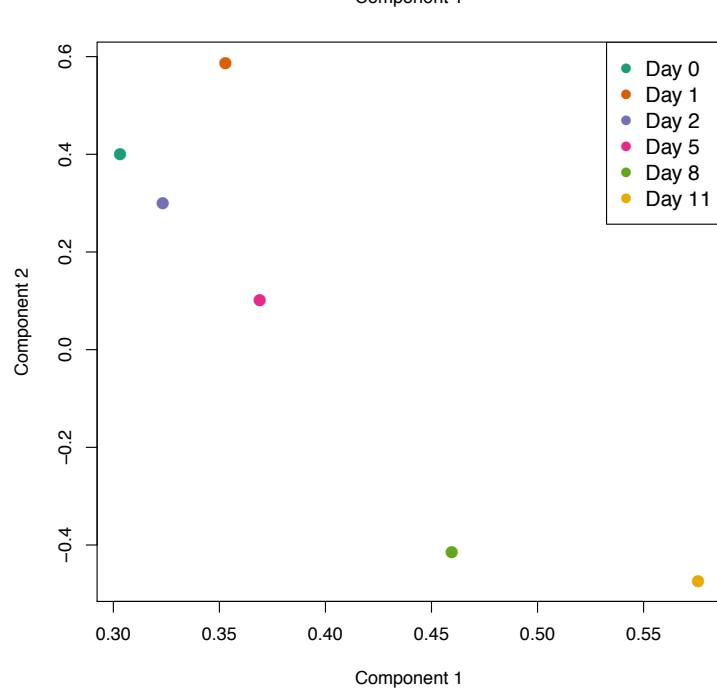

Supplement: Figure S3 — Plot of each sample on the first two components from principal component analysis (PCA) based on gene expression (A), miRNA expression (B) and H3K4me3 levels (C). (PDF) [file pone.0056024.s003.pdf]

NANOG

POU5F1

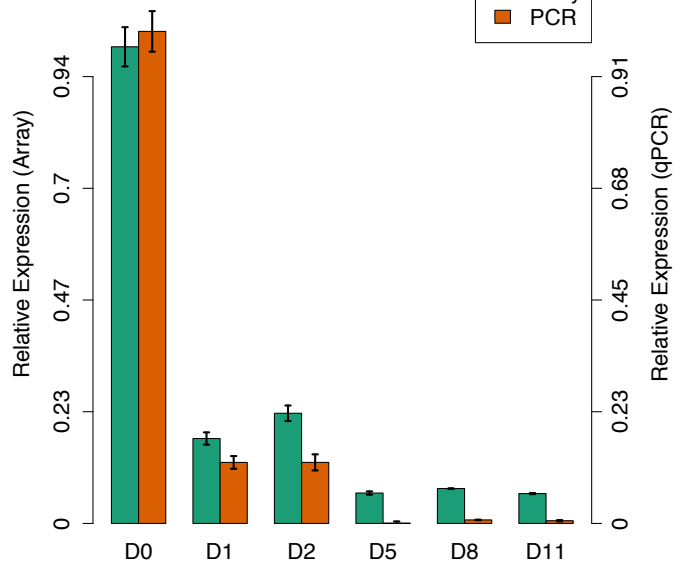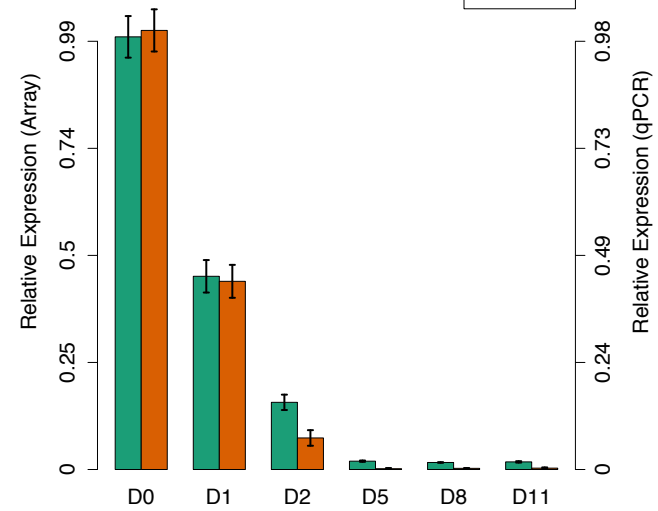

NKX2-2

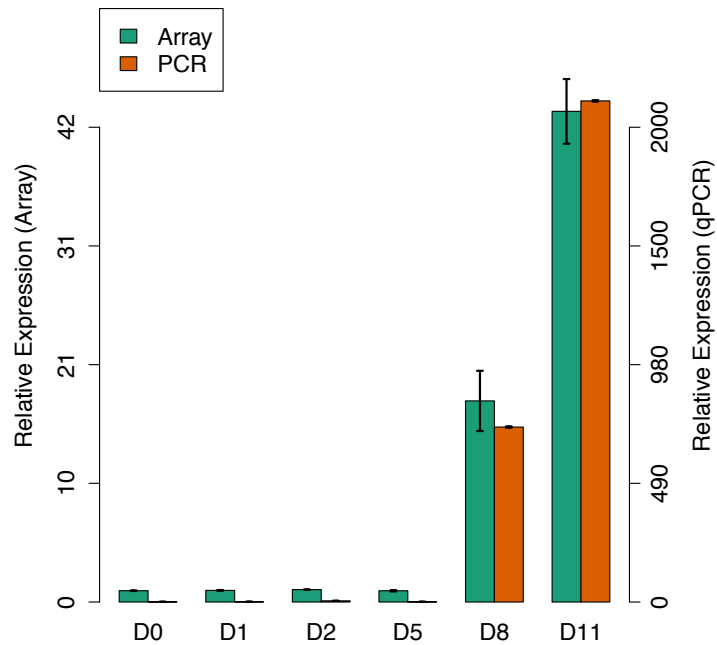

SOX17

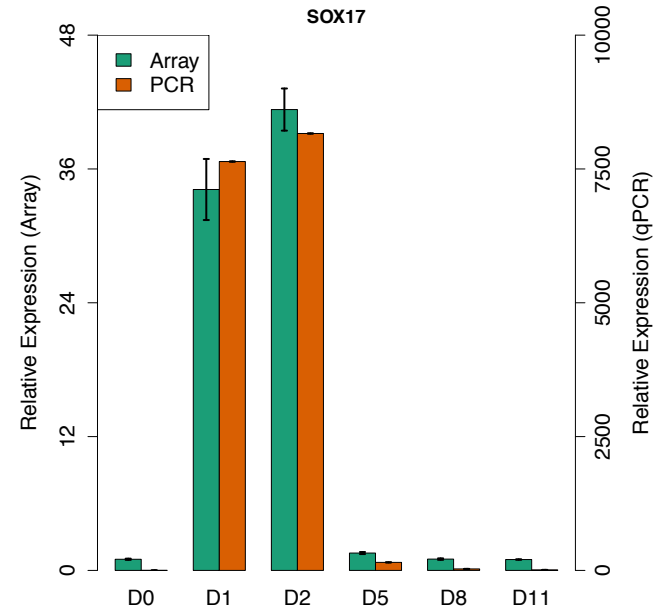

Supplement: Figure S4 — Plot of gene expression data as measured by array (green) and qPCR (orange) for NANOG (A), POU5F1 (B), NKX2-2 (C) and SOX17 (D). All data is normalized such that D0 expression equals 1. (PDF) [file pone.0056024.s004.pdf]

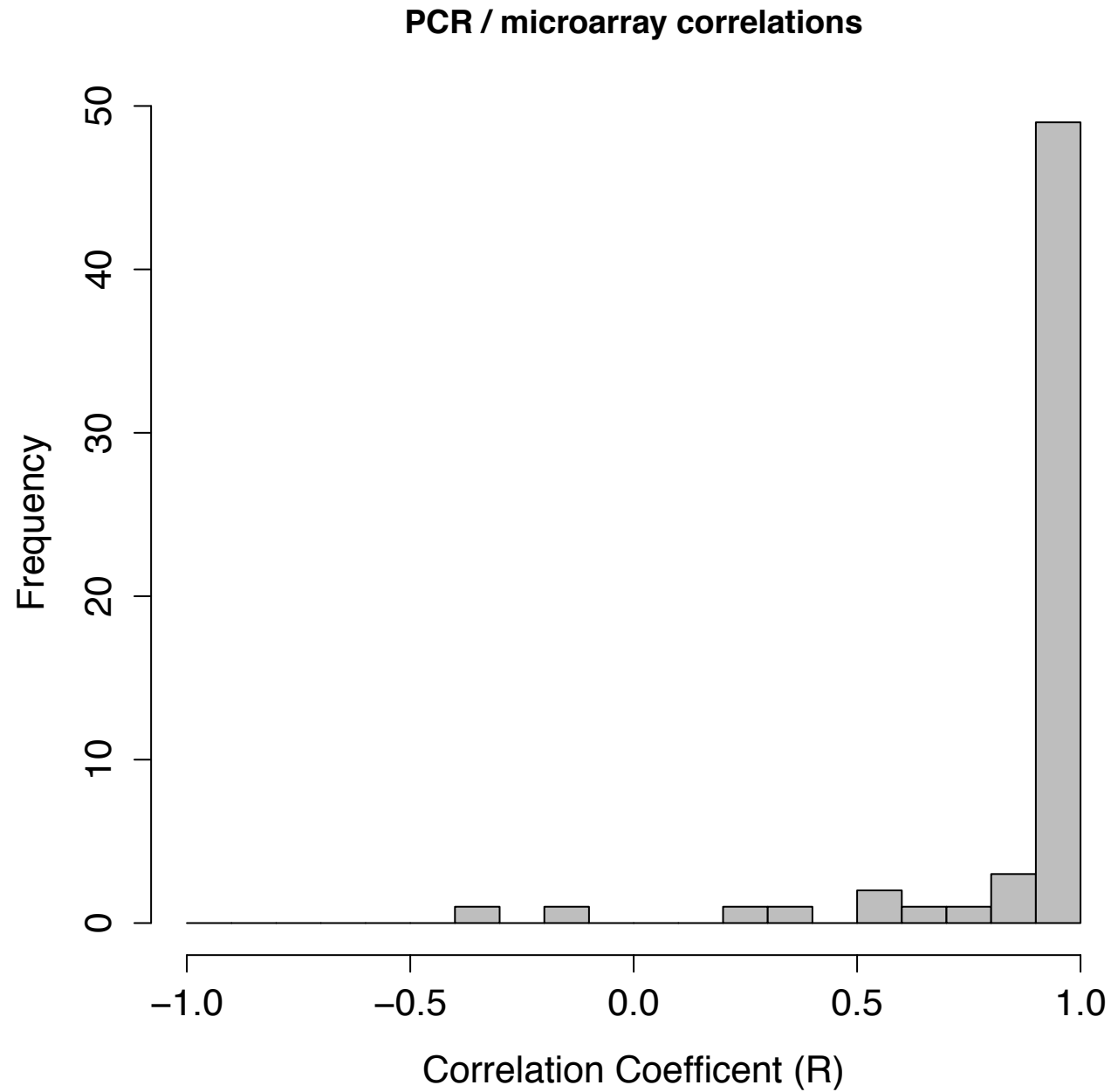

Supplement: Figure S5 — Histogram of the correlation coefficients (R) for each gene between expression levels measured by arrays and qPCR. (PDF) [file pone.0056024.s005.pdf]

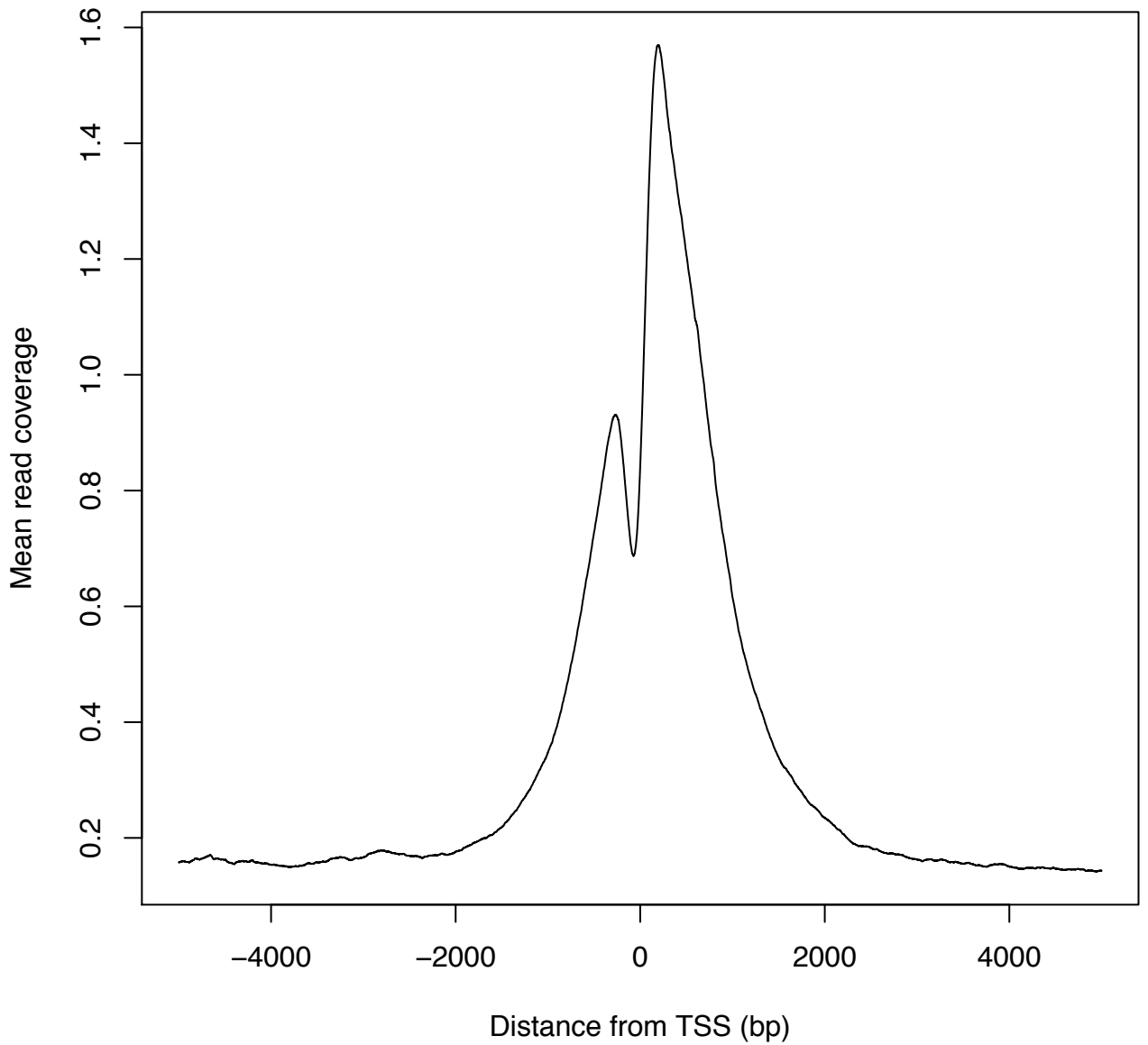

Supplement: Figure S6 — Plot of H3K4me3 read density around transcriptional start sites (TSS) as defined using Ensembl. (PDF) [file pone.0056024.s006.pdf]

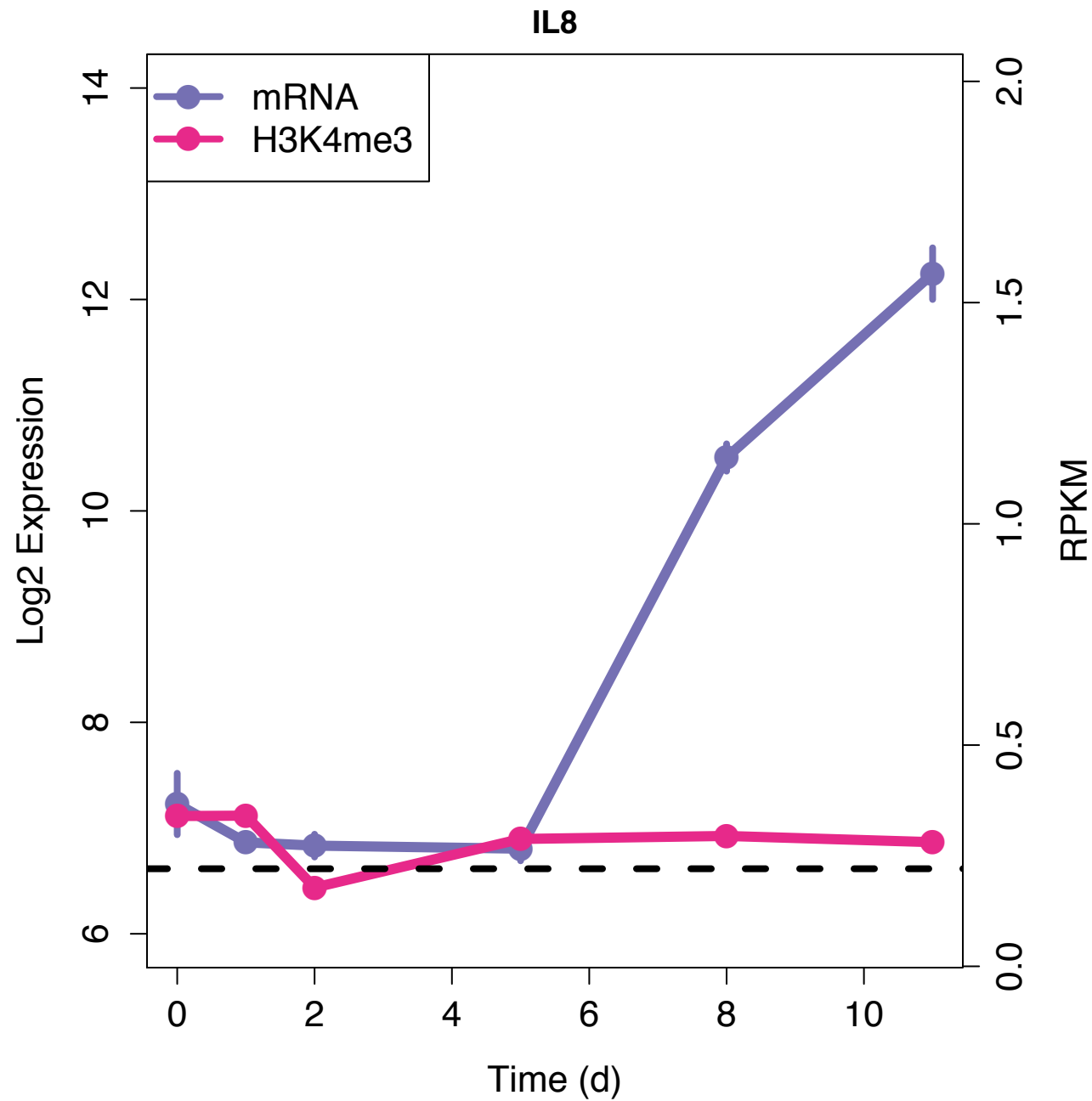

Supplement: Figure S7 — Gene expression (blue) and H3K4me3 levels (red) at each timepoint for IL-8. (PDF) [file pone.0056024.s007.pdf]

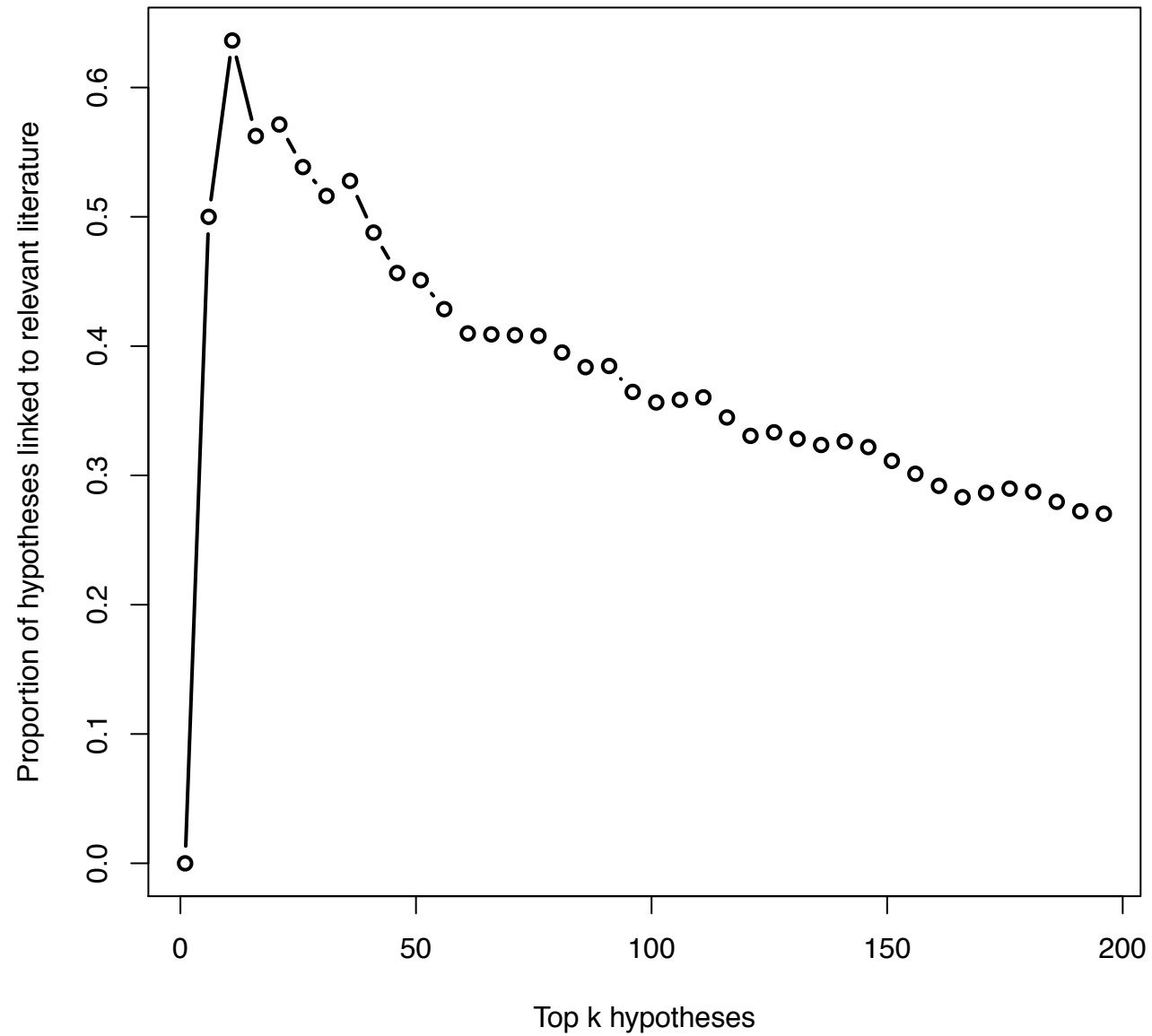

Supplement: Figure S8 — The proportion of CRE hypotheses linked in the literature to beta cell development as a function of the rank cutoff. Genome wide the proportion is ∼5%. FACS of purified endocrine cell population compared to PE aggregates. (PDF) [file pone.0056024.s008.pdf]

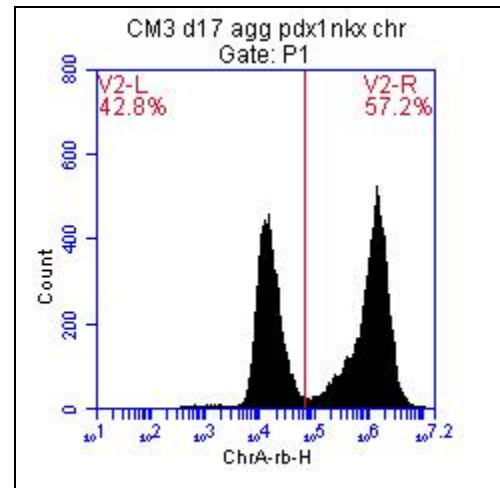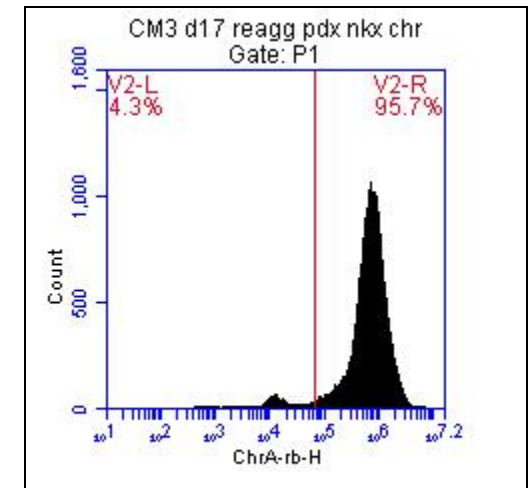

|                                    | Aggregates | Re-Aggregates |
|------------------------------------|------------|---------------|
| <b>Endocrine cells</b> (CHGA+)     | 57.2%      | 95.7%         |
| <b>PE</b> (PDX1+/NKX6.1+/CHGA-)    | 25.0%      | 1.0%          |
| <b>Other</b> (PDX1+/NKX6.1-/CHGA-) | 16.4%      | 2.6%          |

Supplement: Figure S9 — FACS of purified endocrine cell population compared to PE aggregates. (PDF) [file pone.0056024.s015.pdf]
