# Supplementary material for: Novel Pancreatic Endocrine Maturation Pathways Identified by Genomic Profiling and Causal Reasoning
Source: PLoS One. 2013 Feb 13;8(2):e56024. doi: 10.1371/journal.pone.0056024 (PMC3572136; doi:10.1371/journal.pone.0056024)
Supplement: Table S1 — List of 113 genes tested by qRT-PCR. All primer pairs used were human specific except where denoted M for murine specific. (PDF) [file pone.0056024.s009.pdf]

Gene Name	Gene Name	Gene Name	Gene Name
ABCG2	FABP4	IL8	PECAM1
ACAN	Fabp4_M	INS	POU5F1
ACTA2	FGF2	ISL1	PPARG
ACTB	FLT1	KIT	PPY
Actb_M	Flt1_M	KRT18	PROM1
AFP	FOXA2	KRT19	PTF1A
AGGF1	GAPDH	MAFA	PTGS2
Aggf1_M	Gapdh_M	MAFB	PTPRC
ANGPT1	GATA4	MET	REG3A
Angpt1_M	GATA6	MNX1	RFX6
ARX	GCG	MYOD1	RUNX2
B2M	GCK	NANOG	S1PR1
B2m_M	GHRL	NCAM1	S1pr1_M
BHLHA15	GLP1R	NES	SLC2A2
CD34	HES1	NEUROD1	SNAI2
CD44	HGF	NEUROG3	SOX17
CDH1	HHEX	NKX2-2	SOX2
CDH2	HNF1A	NKX6-1	SST
CDH5	HNF1B	NOS2	T
CFTR	HNF4A	NT5E	TEK
CPA1	IAPP	ONECUT1	Tek_M
CSF3	ICAM2	PAX4	TERT
CXCL12	Icam2_M	PAX6	TGFB1
CXCR4	IDO1	PCSK1	THY1
ENG	IGF1	PCSK2	UCHL1
Eng_M	IGF1R	PDGFA	VCAM1
EPCAM	IL10	PDGFRB	VEGFA
EXTL3	IL6	PDX1	Vegfa_M
			VIM

Supplementary Table 1.

List of genes tested by qRT-PCR. All primer pairs used were human specific except where denoted _M for murine specific.
